# *Salmonella typhimurium* co-expressing cytolysin A and hyaluronidase suppresses tumor growth and metastasis

**DOI:** 10.1038/s41420-025-02897-9

**Published:** 2026-01-02

**Authors:** Khuynh Van Nguyen, Dinh-Huy Nguyen, Hien Thi-Thu Ngo, Sung-Hwan You, So-young Kim, Yeongjin Hong, Jung-Joon Min

**Affiliations:** 1 Institute for Molecular Imaging and Theranostics, Chonnam National University Medical School, Hwasun-gun, Jeollanam-do Republic of Korea; 2 Department of Biomedical Science (BrainKorea21 Plus), Chonnam National University Graduate School, Gwangju, Republic of Korea; 3 Department of Nuclear Medicine, Chonnam National University Hwasun Hospital, Hwasun-gun, Jeollanam-do Republic of Korea; 4 Department of Biochemistry, Hanoi Medical University, Dong Da, Hanoi Vietnam; 5 CNCure Co., Ltd., National Immunotherapy Innovation Center, Hwasun-gun, Jeollanam-do Republic of Korea; 6 Department of Microbiology, Chonnam National University Medical School, Gwangju, Republic of Korea

**Keywords:** Targeted therapies, Cell death and immune response

## Abstract

Recently, various attenuated bacteria have been studied as cancer therapies due to their unique characteristics, which include tumor-targeting bioactivity and immunogenicity. Previously, we reported a *Salmonella typhimurium* strain, CNC018, which is attenuated by 10⁵–10⁶-fold compared with the wild-type strain but retains tumor-targeting specificity. However, although these bacteria suppress tumors at the early stage in mice, the tumors often regrow at later stages. Therefore, to increase antitumor efficacy, we used a doxycycline-inducible system to engineer this strain (CNC018pCH) to secrete both cytolysin A (ClyA) and hyaluronidase (HysA), a pore-forming toxin that kills tumor cells and an enzyme that disrupts the tumor microenvironment, respectively. Local secretion of ClyA from CNC018pCH triggered tumor cell death through pyroptosis, apoptosis, and necrosis (PANoptosis) in a cholesterol-dependent manner, thereby releasing cellular contents and danger signals to activate the immune system. In addition, localized secretion of HysA degraded hyaluronic acid secreted by cancer cells, facilitated bacterial penetration into tumors, and inhibited metastasis by down-regulating the ribosomal S6 kinase (RSK)-related signaling pathway. These therapeutic payloads enhanced the ability of *S. typhimurium* to control tumor growth and metastasis in various murine tumor models. Notably, CNC018pCH also generated memory responses by protecting cured mice from tumor rechallenge. Taken together, these findings demonstrate that this engineered bacterium is a promising candidate for cancer treatment by reshaping the tumor microenvironment through the induction of tumor cell death and degradation of hyaluronic acid.

## Introduction

Bacteria-mediated cancer therapy (BCT) is a promising alternative treatment for cancer [1]. To ensure clinical safety, bacteria such as *Salmonella typhimurium*, *Clostridium novyi*, and *Listeria monocytogenes* have been attenuated prior to clinical testing [2].

*S. typhimurium* was attenuated by introducing mutations into various genes related to its pathogenicity. Deletion of the *relA* and s*poT* genes resulted in a nonvirulent strain that was defective in the synthesis of guanosine tetraphosphate (ppGpp), which is an alarmone nucleotide that mediates stringent responses in bacteria by inhibiting RNA synthesis under conditions of amino acid starvation [3]. It also affects bacterial physiology and virulence, including stress responses, regulation of virulence factors, antibiotic tolerance, and biofilm formation [4]. A strain deficient in ppGpp biosynthesis (i.e., ΔppGpp) is attenuated, with a lethal dose 50 (LD_50_) in mice that is 10⁵–10⁶-fold higher than that of the wild-type strain [2]. Nevertheless, this strain retains its tumor-targeting ability and therapeutic efficacy in tumor-bearing mice by increasing the secretion of proinflammatory cytokines and by activating inflammasomes [5]. We further attenuated the ΔppGpp strain by removing Salmonella pathogenicity islands 1 and 2 (*SPI-1* and *SPI-2*), gene clusters involved in bacterial invasion of host cells and in intracellular survival and proliferation, respectively [6]. The resulting *S. typhimurium* strain CNC018 (ΔppGpp, ΔSPI-1, ΔSPI-2) is more attenuated than the ΔppGpp *S. typhimurium* strain [7].

Despite these efforts to develop safe bacterial therapies, in mice receiving BCT with this bacterium, tumors are suppressed strongly at the early stage, but often regrow at later stages [5]. This indicates that the immune response triggered by attenuated *S. typhimurium* is not sufficiently strong or long-lived to achieve complete antitumor efficacy. However, co-administration of antitumor drugs such as immune checkpoint blockers improves BCT efficacy [8]. Another approach is to engineer bacteria to express antitumor payloads such as toxins, cytokines, and immune modulators [1, 2, 9]. Among these, cytolysin A (ClyA) is a promising candidate. This toxin, originally secreted by *S. typhi* via the Type 10 secretion system, is incorporated into outer membrane vesicles (OMVs) [10]. These OMVs facilitate the transport of ClyA to the extracellular space, enabling it to efficiently form pores on the surface of target tumor cells that overexpress cholesterol [11]. *S. typhimurium* secreting ClyA induces significantly better antitumor immune responses in treated mice [12]. The tumor-targeting ability of bacteria minimizes potential toxic effects in normal organs, although the toxin can bind to cholesterol on both tumor and normal cells [13].

When *S. typhimurium* targets tumor tissue, it becomes confined to hypoxic areas due to a neutrophil barrier [14], which prevents bacteria from disseminating throughout the entire tumor tissue, resulting in insufficient antitumor effects. Depleting neutrophils using antibodies disrupts this barrier, allowing for wider bacterial dispersion and stronger antitumor effects [14]. This suggests that disrupting the complexity of the tumor microenvironment (TME) is crucial for bacterial dispersion. Hyaluronic acid (HA), a major component of the extracellular matrix [15], promotes cell proliferation and migration, and is involved in the progression of many malignant tumors [16]. Degrading HA by hyaluronidase (HysA), an enzyme with an N-terminal signal peptide that likely facilitates its translocation into the periplasm via the general Sec (secretion) pathway [17], disrupts the TME [18], resulting in enhanced antitumor efficacy of chemotherapy in preclinical trials [19]. Notably, recent studies have demonstrated that HysA-mediated HA degradation enhances *S. typhimurium* penetration into tumor tissues and reduces the metastatic potential of cancer cells [20–22]. This dual effect of modulating the TME—improving bacterial distribution for enhanced delivery of antitumor payloads directly to tumor tissues and suppressing tumor cell metastasis—highlights its potential to optimize *S. typhimurium*-based cancer therapies.

**Fig. 1:**
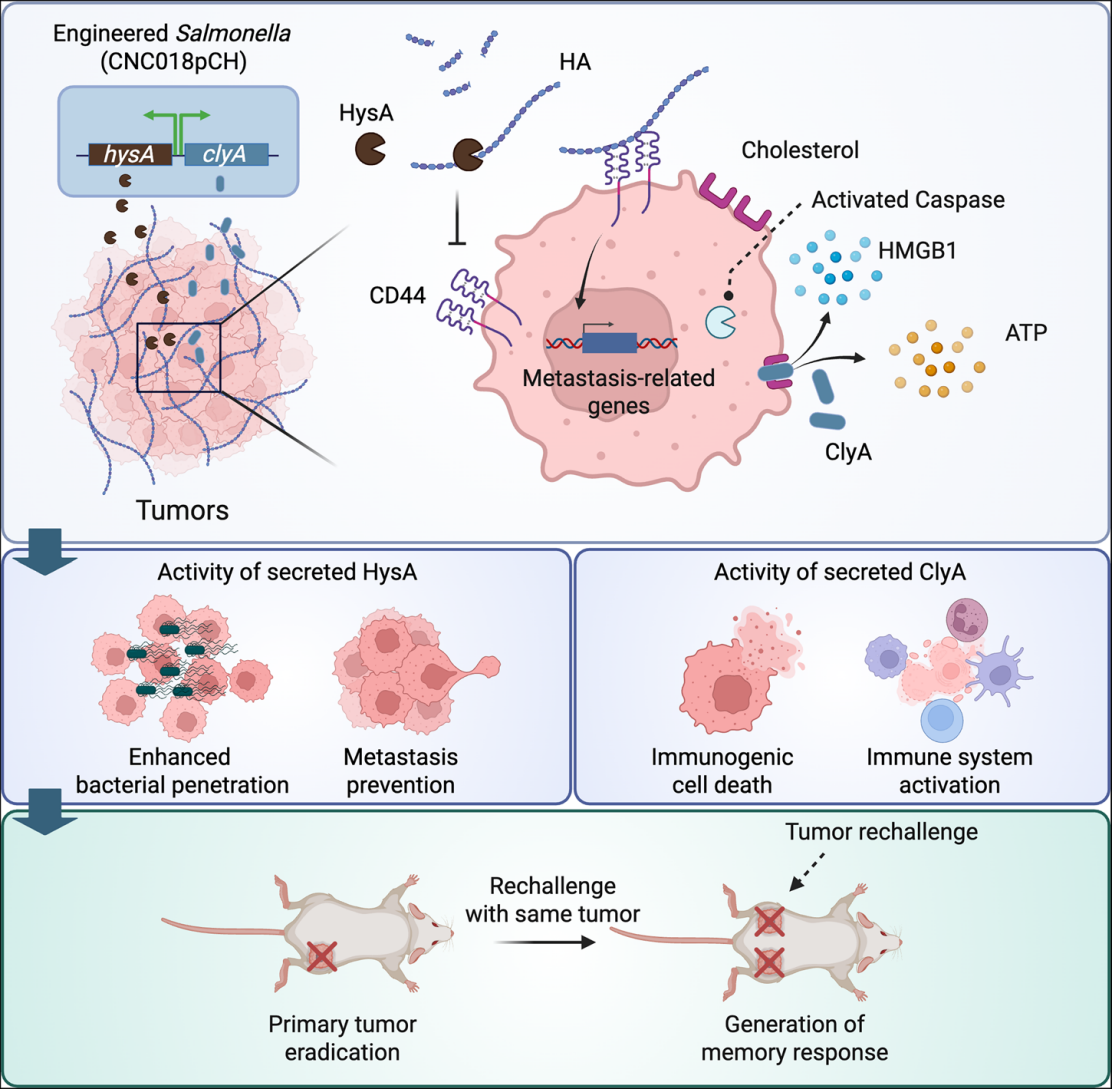
Schematic illustration of an engineered *Salmonella* co-secreting ClyA and HysA for cancer treatment. The figure was created using BioRender and has confirmed publishing and licensing rights (agreement number EI292XOGXB). Based on the natural tumor-targeting ability of *S. typhimurium* CNC018, the strain was engineered (CNC018pCH) to locally secrete dual antitumor payloads, HysA and ClyA. HysA facilitates bacterial penetration and helps prevent metastasis, while ClyA induces tumor cell death through PANoptosis and activates the immune system. Consequently, CNC018pCH has the potential to control tumor growth and metastasis while generating long-term antitumor immune memory.

Here, we engineered an attenuated *S. typhimurium*, CNC018pCH, to secrete both ClyA and HysA directly into tumor tissues under the control of a doxycycline (Doxy) inducer without compromising bacterial growth. We subsequently evaluated its antitumor efficacy and capacity to prevent metastasis in mice bearing various types of tumors (Fig. 1).

## Results

### Engineering of *S. typhimurium* (CNC018pCH) to co-express ClyA and HysA

**Fig. 2:**
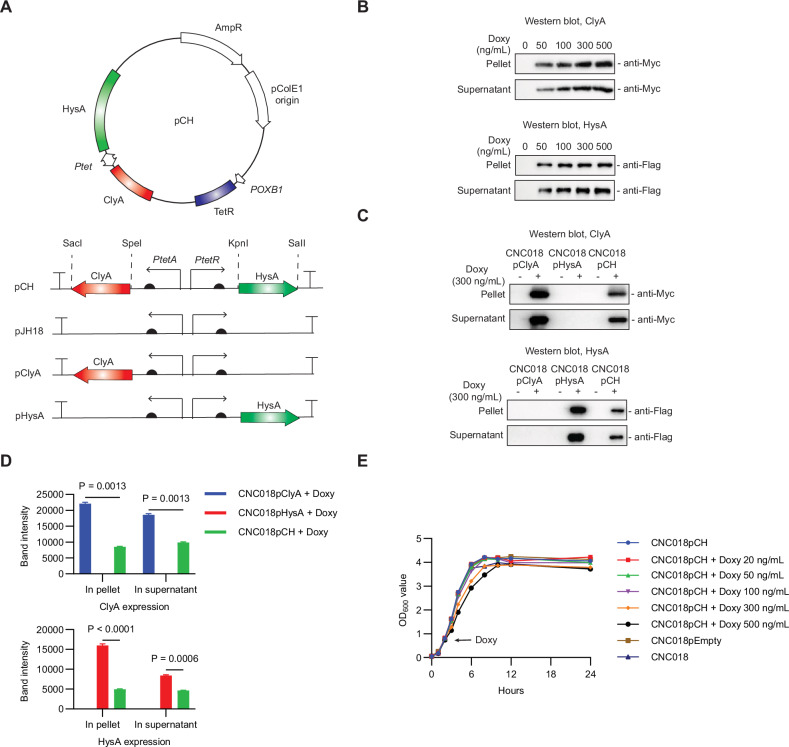
Establishment of *S. typhimurium* CNC018 secreting ClyA and HysA. **A** Schematic representation of the pCH plasmid. The genes encoding ClyA and HysA are under control of the *PtetA* and *PtetR* promoters, respectively, in plasmid pJH18 (referred to as pCH), and are induced by Doxy (upper panel). The locations of payload genes and restriction sites are illustrated, along with the promoters in pCH, and in plasmids pClyA and pHysA, which carry the *clyA* and *hysA* genes, respectively (bottom panel). AmpR ampicillin resistance gene, TetR tetracycline repressor gene. **B** Western blot analysis of ClyA and HysA expression by CNC018pCH. After Doxy induction during the mid-log phase, bacterial pellets and supernatants were collected after a 5-h culture and subjected to western blot analysis with anti-Myc-tag and anti-Flag-tag antibodies to detect ClyA and HysA, respectively. **C** Western blot analysis of ClyA and HysA expression by CNC018 carrying different plasmids. After Doxy induction (300 ng/mL for 5 h), bacterial pellets and supernatants were analyzed by western blotting with anti-Myc-tag and anti-Flag-tag antibodies. **D** Quantification of the intensity of the bands in (**C**) using ImageJ software. The data are represented as mean ± s.e.m. from three different experiments. Statistical significance was assessed by two-way ANOVA with Tukey’s multiple comparisons test. **E** Growth curve of CNC018pCH in the presence of Doxy. CNC018pCH was cultured in fresh ampicillin-containing LB medium, and Doxy was added during the mid-log phase (OD_600_ 0.5–0.7, as indicated by the arrow). Bacterial growth was assessed by measuring OD_600_ values at the indicated times.

First, the genes encoding the payloads ClyA and HysA were cloned into the pJH18 plasmid behind a Doxy-inducible dual *Ptet* promoter (*PtetA/PtetR*) [23]; the resulting plasmid was named pCH (Fig. 2A). The cloning was confirmed by PCR analysis, restriction mapping (Fig. S1A and B), and sequencing. Subsequently, the pCH plasmid was transformed into the attenuated *S. typhimurium* strain CNC018 (ΔppGpp, ΔSPI-1, ΔSPI-2), which was designated CNC018pCH.

Next, we assessed payload expression in CNC018pCH treated with Doxy. Both ClyA and HysA were detected after treatment with 50 ng/mL Doxy, with maximum expression at 300 ng/mL Doxy, as shown by western blot analysis of the bacterial pellet and culture supernatant (Fig. 2B; the full-length, uncropped western blots are provided in Supplementary Original Western Blots). This indicates that expression and secretion were effectively regulated by Doxy. We also confirmed that CNC018 harboring plasmids carrying only the *clyA* or *hysA* gene (CNC018pClyA or CNC018pHysA) expressed their respective proteins at 300 ng/mL Doxy, similar to CNC018pCH (Fig. 2C, D). Notably, the protein levels were quite high, even higher than those in CNC018pCH. This may be due to the metabolic burden of the plasmid on protein production [24]. Although the levels of secreted ClyA and HysA in CNC018pCH were approximately 2-fold lower than those in CNC018pClyA and CNC018pHysA, respectively, we aimed to investigate whether the combined effect of ClyA and HysA was superior to that of each cargo alone.

To examine whether payload expression affected bacterial growth, CNC018pCH was cultured overnight at 37 °C in the presence of various concentrations of Doxy. Growth was affected only slightly by Doxy-induced payload expression (Fig. 2E). In addition, the bacterial intracellular protein DnaK was not detected significantly in the CNC018pCH supernatant at 300 ng/mL Doxy (Fig. S1C), indicating that Doxy at 300 ng/mL does not significantly affect bacterial growth.

### ClyA kills cancer cells via the PANoptosis pathway

**Fig. 3:**
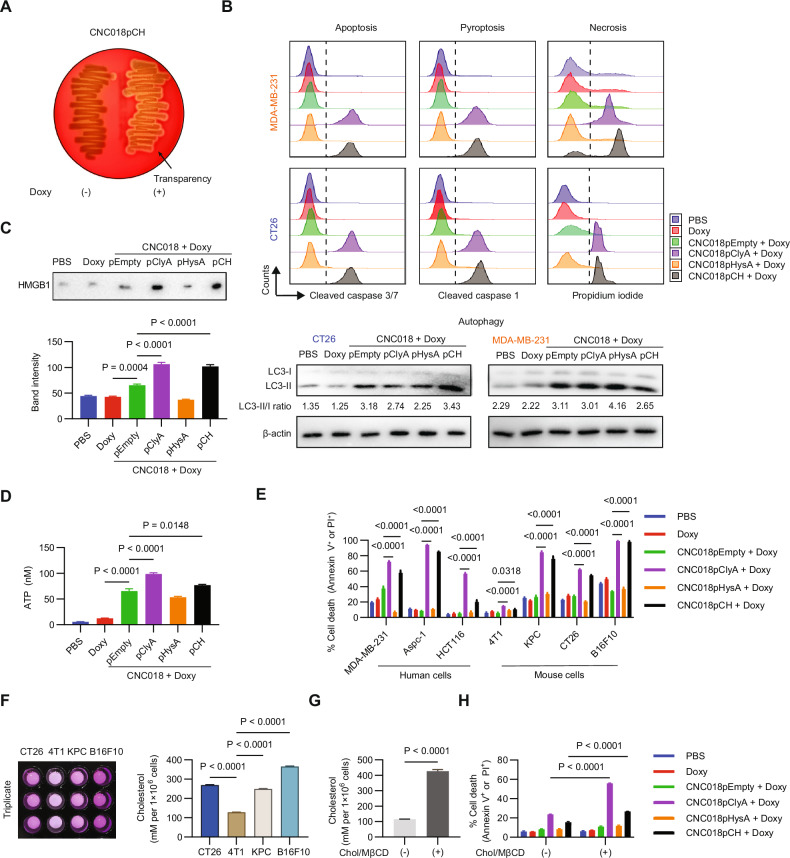
CNC018pCH-induced PANoptotic cancer cell death and release of danger signals. **A** ClyA activity of CNC018pCH. CNC018pCH was streaked onto a blood agar plate with or without 300 ng/mL Doxy and incubated overnight at 37 °C. Transparent zones around colonies indicate ClyA-induced hemolysis. **B** Flow cytometry and western blot analysis of specific cell death markers in tumor cells treated with bacteria and Doxy. MDA-MB-231 and CT26 cells (3 × 10⁵) were seeded in 12-well plates. After overnight culture, the cells were treated with bacteria (1 MOI) plus 300 ng/mL Doxy and then incubated for 16 h. Cells were then collected and stained with antibodies specific for cleaved caspases 3 and 7 (apoptosis), cleaved caspase-1 (pyroptosis), and propidium iodide (PI; necrosis), followed by flow cytometry analysis. Autophagy in cancer cells was assessed by western blotting with an anti-LC3 antibody. Conversion of LC3-I to LC3-II indicates autophagy. **C**, **D** Release of danger signals from tumor cells treated with ClyA-secreting bacteria. CT26 tumor cells (3 × 10⁵) were treated with bacteria and 300 ng/mL Doxy. After 16 h, levels of DAMP markers HMGB1 and ATP were measured in the culture supernatant by western blotting to detect HMGB1 and using an ATP assay kit. **C** Western blot analysis of HMGB1, with band intensity quantified by ImageJ software. **D** ATP levels in the culture supernatant. **E** Cytotoxicity of CNC018pCH against different cancer cell types. Cells (3 × 10⁵) were seeded in 12-well plates, treated with bacteria (1 MOI) plus 300 ng/mL Doxy, and then incubated for 16 h. Cells were then stained with Annexin V-PI and analyzed by flow cytometry. **F** Cholesterol levels in tumor cells. Cholesterol was extracted from 1 × 10⁶ cells and measured using a cholesterol assay kit. **G** Cholesterol levels in 4T1 cells after cholesterol supplementation. 4T1 cells (1 × 10⁶) in 6-well plates were pretreated with 10 mM MβCD for 60 min, followed by exposure to 100 μM cholesterol in methyl-β-cyclodextrin for 12 h. Cells were then collected for cholesterol measurement. **H** Cytotoxicity of CNC018pCH against 4T1 tumor cells supplemented with cholesterol. Cholesterol-supplemented 4T1 tumor cells were incubated with bacteria (MOI 1) plus 300 ng/mL Doxy for 16 h. Cells were then stained with Annexin V-PI and analyzed by flow cytometry. Data are shown as mean ± s.e.m. from three independent experiments. Statistical significance was determined using one-way ANOVA (**C**, **D**, **F**), two-way ANOVA (**E**, **H**), or unpaired two-tailed t-test (**G**), with Tukey’s multiple comparisons test applied where appropriate.

To assess whether ClyA remained functional after secretion, CNC018pCH bacteria were cultured on red blood cell agar with or without 300 ng/mL Doxy. Transparent areas appeared around the colonies only on plates containing Doxy, indicating that the bacteria secreted functional ClyA (Fig. 3A).

ClyA induces cell death via apoptosis or necrosis [11, 25–27]. To address the previously inconsistent findings about the role of ClyA role in cell death, we stained markers of different cell death pathways in human MDA-MB-231 and mouse CT26 tumor cells treated with bacteria and Doxy (Fig. 3B). Only ClyA-secreting bacteria (CNC018pCH and CNC018pClyA) showed a strong increase in expression of markers of pyroptosis (cleaved caspase-1), apoptosis (cleaved caspase-3/7), and necrosis (PI), whereas there was no significant difference in markers of autophagy (enhanced LC3-II/LC3-I ratio), compared with CNC018pEmpty and CNC018pHysA. These findings indicate that ClyA forms pores in tumor cells, leading to PANoptosis (P: pyroptosis, A: apoptosis, N: necrosis). Consequently, tumor cells underwent rapid rupture and released their intracellular contents. Consistent with this, intracellular damage-associated molecular patterns (DAMPs) such as ATP, HMGB1, and the intracellular enzyme LDH were found in significantly higher amounts in the culture supernatant of CT26 tumor cells treated with ClyA-secreting bacteria (CNC018pCH and CNC018pClyA) (Fig. 3C, D, and Fig. S2).

### CNC018pCH kills cancer cells in a cholesterol-dependent manner

To evaluate ClyA activity against other cancer cell types, seven different tumor cell lines were treated with bacteria plus 300 ng/mL Doxy, followed by staining with Annexin V and PI after 16 h (Fig. 3E). ClyA-secreting bacteria (CNC018pCH and CNC018pClyA) increased cell death significantly in all tested tumor cell lines (except 4T1) compared with controls. We hypothesized that this was related to cholesterol levels in tumor cells, as ClyA is known to bind to cholesterol in the cell membrane to form pores [13]. Supporting this hypothesis, cholesterol levels in 4T1 cells were >2-fold lower than those in CT26, KPC, and B16F10 cells (Fig. 3F). Furthermore, enriching 4T1 cells with cholesterol dissolved in methyl-β-cyclodextrin (Chol/MβCD) [13] increased cell death induced by CNC018pCH significantly (Fig. 3G, H, and Fig. S3).

### HA levels correlate with the metastatic capacity of cancer cells

**Fig. 4:**
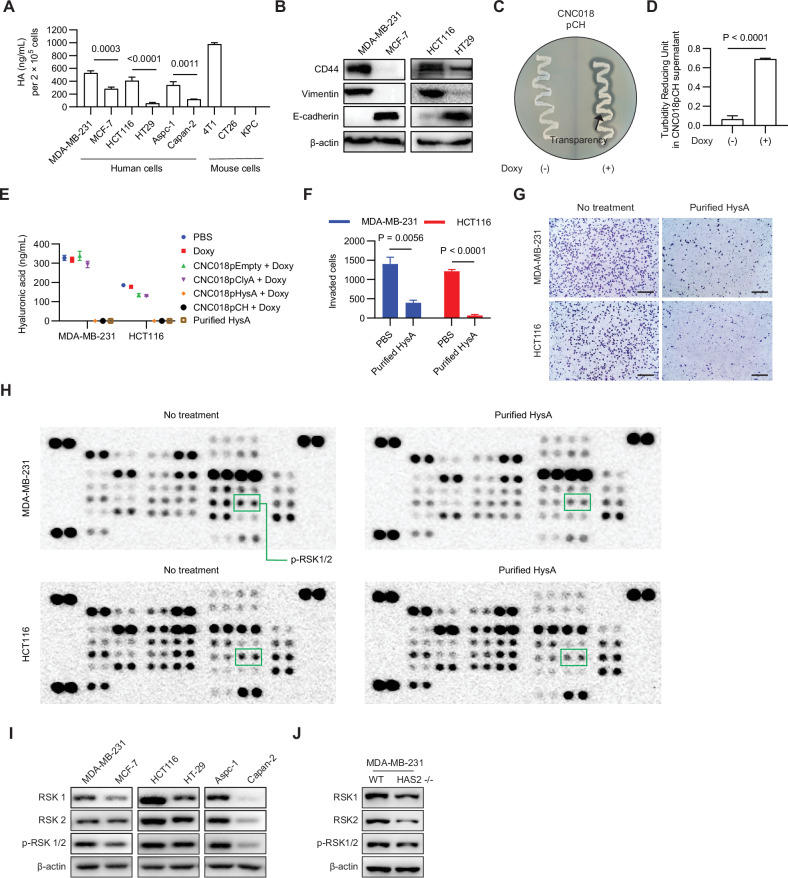
Remodeling of the tumor extracellular matrix and suppression of metastasis-related signaling by CNC018pCH-derived HysA. **A** Quantification of HA levels in cell culture medium. Cells (2 × 10⁵) were seeded in 12-well plates. After 24 h, the culture medium was collected and analyzed using an HA quantification kit. **B** Expression of CD44, the mesenchymal marker vimentin, and the epithelial marker E-cadherin by different cancer cell types. Cell lysates (20 µg) were subjected to western blot analysis with antibodies against CD44, vimentin, and E-cadherin. β-actin was used as a loading control. **C** HysA activity of CNC018pCH. CNC018pCH was streaked onto an HA agar plate with or without 300 ng/mL Doxy and incubated overnight at 37 °C. HA acid-degraded clear zones were observed after acetic acid treatment. **D** Quantification of HysA activity in CNC018pCH supernatants. After Doxy induction (300 ng/mL for 5 h), bacterial supernatants were analyzed in an HysA activity assay. Activity was expressed as turbidity reduction units (TRU). **E** HA degradation in cell culture media by HysA-secreting bacteria. 2 × 10⁵ MDA-MB-231 or HCT116 cells were treated with the supernatants of engineered CNC018 strains for 24 h. 100 units/mL of purified HysA was used as a positive control. The concentration of HA was measured using an ELISA kit. **F** Invasion assay. Cells (3 × 10⁴/well) were seeded in 24-well Matrigel invasion chambers and treated with 100 units/mL purified HysA for 24 h. Invaded cells were stained and counted under a light microscope. **G** Representative images of the invasion assay in (**F**). Scale bar: 100 μm. **H** Phospho-kinase array analysis. MDA-MB-231 or HCT116 cells (3 × 10⁵/well) were seeded in 12-well plates and treated with 100 units of purified HysA for 24 h. Cell lysates (200 µg protein) were analyzed using a phospho-kinase array kit (R&D Systems, ARY003C). Please also see the phospho-kinase array coordinate map in Fig. S6. **I** Phospho-kinase expression in high and low metastatic cancer cells. Cell lysates (20 µg) were analyzed by Western blotting with anti-RSK antibodies. **J** Phospho-kinase expression in HA-downregulated MDA-MB-231 cells. The *HAS2* gene was knocked out using CRISPR/Cas9. Cell lysates (20 µg) were analyzed by western blotting with anti-RSK antibodies. WT: wild-type MDA-MB-231 cells; HAS2⁻/⁻: *HAS2*-knockout MDA-MB-231 cells. Data are presented as mean ± s.e.m. from three independent experiments. Statistical analyses were performed using one-way ANOVA (**A**), unpaired two-tailed t-test (**D**), two-way ANOVA (**E**, **F**), with Tukey’s post-hoc test where applicable.

HA is a major component of the extracellular matrix in tumor tissues [28], and its levels are related to the metastatic capacity of tumor cells [29]. Consistent with this, expression of HA by human tumor cells with high metastatic capacity (MDA-MB-231, HCT116, and Aspc-1) was relatively higher than that by others (MCF7, HT29, and Capan-2) (Fig. 4A and Fig. S4A). Usually, expression of the mesenchymal marker vimentin is increased, and that of the epithelial marker E-cadherin is decreased, in highly metastatic cells [30]. We confirmed that this was the case by conducting Western blot analysis and flow cytometry. As expected, vimentin was more commonly expressed at higher levels in human tumor cells with high metastatic capacity (Pearson’s correlation coefficient, r = 0.886, p = 0.0015), and E-cadherin was minimally expressed in cells with high metastatic capacity (r = -0.7135, p = 0.0309) (Fig. 4B, Fig. S4B, C). Taken together, these findings indicate that expression of HA and CD44 correlates with the metastatic capacity of cancer cells.

### Role of HysA in preventing metastasis

To assess the HysA enzyme activity in transformed bacteria, we cultured CNC018pCH on HA agar plates with or without 300 ng/mL Doxy. Transparent zones formed around the colonies only on Doxy-containing plates, indicating that the bacteria secreted functional HysA (Fig. 4C). Next, we measured HysA enzyme activity in the supernatant of CNC018pCH cultured at 300 ng/mL Doxy using the HysA activity assay. After 45 min of incubation, activity was clearly observed only in supernatants of HysA-secreting bacteria (CNC018pCH and CNC018pHysA) (Fig. S5). The HysA activity of CNC018pCH increased by 7.7-fold upon Doxy induction (Fig. 4D).

To investigate the effects of HysA on tumor cells, highly metastatic tumor cells (MDA-MB-231, HCT116) were treated with the supernatants of engineered CNC018 strains or with purified HysA (100 units/mL) from CNC018pCH. Both CNC018pCH and CNC018pHysA, as well as the purified HysA, effectively degraded HA in the cell culture medium (Fig. 4E).

Since CD44 plays a critical role in tumor cell invasion, we performed an invasion assay after HA degradation in MDA-MB-231 and HCT116 cells. We observed that purified HysA reduced invasion by both cell lines (Fig. 4F and G). This suggests that HA contributes to tumor cell invasion, and that the functions of CD44 during invasion and metastasis are limited in the absence of HA. To further elucidate HA signaling during tumor cell invasion, we conducted a phospho-kinase array specific for 37 kinases (Fig. 4H and Fig. S6). The results revealed that HysA decreased the phosphorylation of RSK1/2 in both MDA-MB-231 and HCT116 cells. To further investigate the link between RSKs and tumor cell invasion, we compared the expression of total and phosphorylated RSK1 and RSK2 proteins between high and low metastatic cancer cells. As expected, we found that highly metastatic tumor cells expressed higher levels of total and phosphorylated RSK1 and RSK2 proteins (Fig. 4I). These findings suggest that RSK plays roles in tumor cell invasion. To confirm the relationship between RSKs and HA levels, we knocked out the *HAS2* gene encoding the HA-producing enzyme in MDA-MB-231 cells (Fig. S7) [31], and found that in HA-downregulated MDA-MB-231 cells, total and phosphorylated RSKs were downregulated (Fig. 4J). These findings indicate that degradation of HA by HysA can downregulate RSK signaling in various tumor cells, thereby inhibiting invasion and metastasis.

### Optimizing the Doxy dose for in vivo treatment

**Fig. 5:**
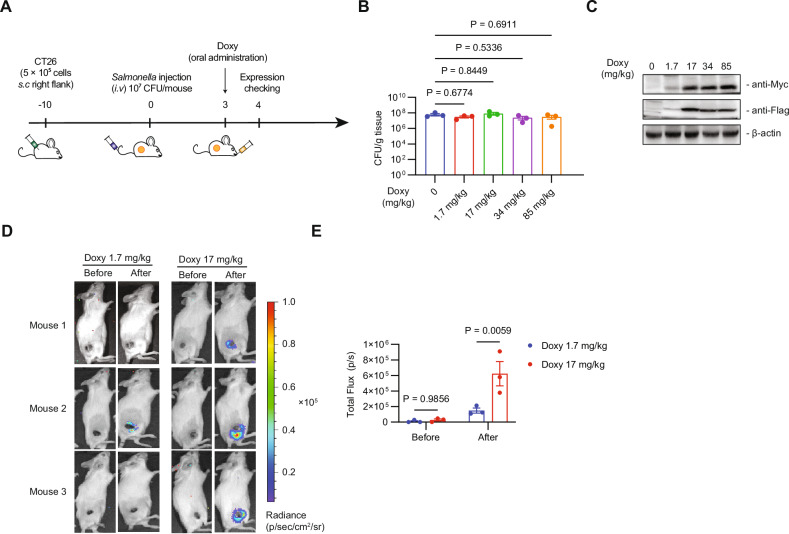
In vivo optimization of therapeutic payload production by engineered CNC018pCH. **A** Experimental scheme. CT26 cells (5 × 10⁵) were injected subcutaneously into the right flank of mice (three mice per group). When the tumor volume reached 100–120 mm³, the mice were injected with bacteria (1 × 10⁷ CFU), followed by oral administration of the indicated amounts of Doxy on Day 3. After 24 h, tumors were harvested. **B** Localization of CNC018pCH in tumors. The number of CNC018pCH bacteria in the tumors was quantified. **C** Western blot analysis. Tumor samples (40 µg protein) were subjected to western blot analysis with Myc-tag (for ClyA detection) or Flag-tag (for HysA detection) antibodies. **D** Luminescence imaging of CNC018pRluc8, with Doxy administered at 1.7 mg/kg (n = 3) and 17 mg/kg (n = 3). The experiment followed the same procedure as in (**A**). Twelve hours after Doxy administration, mice were injected intravenously with coelenterazine (0.7 mg/kg), and imaging was performed with an IVIS machine. **E** Quantification of bioluminescence signals. The signals in (**D**) were quantified. Signal intensity was expressed as units of total flux per second. Data are presented as mean ± s.e.m. Statistical analyses were performed using one-way ANOVA (**B**) or two-way ANOVA (**E**) with Tukey’s multiple comparisons test.

Previously, we demonstrated that *S. typhimurium* selectively targets tumors in mice [23]. To determine the optimal dose of Doxy required for induction, we injected CNC018pCH intravenously into CT26 tumor-bearing mice, and administered Doxy on Day 3 (Fig. 5A). Then number of tumor-localizing bacteria did not change significantly on Day 4 across all Doxy conditions (0–85 mg/kg), indicating that these doses of Doxy do not affect survival of CNC018pCH (Fig. 5B) or lead to plasmid loss (Fig. S8). Expression of ClyA and HysA was detected at 1.7 mg/kg Doxy, reaching its peak at 17 mg/kg Doxy (Fig. 5C). This result is consistent with that observed for CNC018pRluc8, a transformant carrying pRluc8 containing the *rluc8* gene in pJH18. Bioluminescence signals were stronger in CT26 tumor tissues treated with 17 mg/kg Doxy, weaker in those treated with 1.7 mg/kg Doxy, and undetectable in the absence of Doxy (Fig. 5D and E).

### CNC018pCH effectively suppresses tumor growth and metastasis in mice

**Fig. 6:**
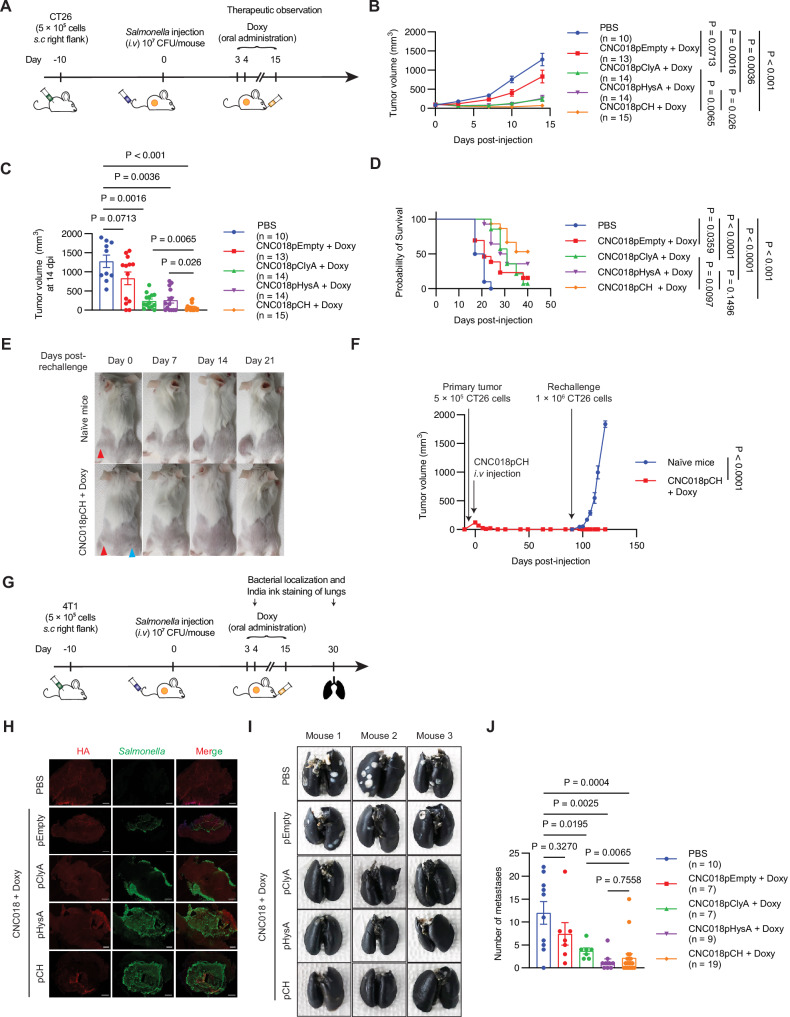
CNC018pCH-mediated induction of tumor regression, metastasis inhibition, and long-term antitumor memory in tumor-bearing mouse models. **A** Experimental scheme. CT26 cells (5 × 10⁵) were injected subcutaneously into the right flank of mice. Once the tumor size reached 100–120 mm³, bacteria (1 × 10⁷ CFU) were injected intravenously, followed by oral administration of 17 mg/kg Doxy every day starting from Day 3 post-infection. **B** Growth curves for CT26 tumors in tumor-bearing mice. PBS (n = 10), CNC018pEmpty + Doxy (n = 13), CNC018pClyA + Doxy (n = 14), CNC018pHysA + Doxy (n = 14), and CNC018pCH + Doxy (n = 15). Tumor growth curves for individual mice are shown in Fig. S9A. **C** Tumor volume at Day 14 post-treatment. **D** Kaplan–Meier survival curve of CT26 tumor-bearing mice. **E** Representative images of CT26 tumor-eradicated mice after rechallenge with CT26 tumors. BALB/c mice were implanted with CT26 cells (5 × 10⁵). CNC018pCH was intravenously injected, followed by Doxy administration. Mice whose tumors were eradicated after CNC018pCH + Doxy treatment were subcutaneously rechallenged with CT26 tumor cells on day 90. Blue triangles, initial CT26 tumors; red triangles, rechallenged CT26 tumors. **F** Average growth curves after CT26 tumor rechallenge. CNC018pCH + Doxy (n = 3) and naïve mice (n = 3). **G** Experimental scheme. 4T1 cells (5 × 10⁵) were injected subcutaneously into the right flank of mice. Bacteria (1 × 10⁷ CFU) were injected intravenously, followed by oral administration of 17 mg/kg Doxy every day starting from Day 3 post-infection. **H** Detection of HA and CNC018 in 4T1 tumor tissues. Once the tumor volume reached 100–120 mm³, mice were injected with bacteria and subsequently administered an oral dose of 17 mg/kg Doxy on Day 3. On Day 4, tumor tissues were stained with an anti-*S. typhimurium* antibody and an HA-binding protein to visualize the presence of CNC018 and HA, respectively. Scale bar: 1 mm. **I** Representative images of 4T1 lung metastases after treatment with bacteria. Mice bearing subcutaneous 4T1 tumors underwent the same treatment as in (**G**). On Day 30, lungs were stained with India ink. **J** Quantification of 4T1 metastatic nodules. PBS (n = 10), CNC018pEmpty + Doxy (n = 7), CNC018pClyA + Doxy (n = 7), CNC018pHysA + Doxy (n = 9), and CNC018pCH + Doxy (n = 19). Data are presented as mean ± s.e.m. from two independent experimental replicates. Statistical analyses were performed using one-way ANOVA with Tukey’s multiple comparisons test (**B**, **C**, **F**, **J**) or log-rank (Mantel–Cox) test (**D**).

Next, we investigated the therapeutic effect of CNC018pCH in mice bearing murine colon tumors (CT26). Both CNC018pHysA and CNC018pClyA showed antitumor effects, but CNC018pCH was the most potent (Fig. 6A–D, Fig. S9A, B). CNC018pCH also increased the survival time of mice, extending the median survival time after treatment from 19 days in the PBS group to 41 days (a 116% increase) in the CNC018pCH treatment group. Furthermore, there were no significant changes in the appearance or body weight of mice after treatment (Fig. S9C). Finally, mice that remained tumor-free for 90 days after treatment were rechallenged by injecting twice the original number of CT26 cells into the opposite flank (Fig. 6E and F). Notably, unlike age-matched naïve controls, the cured mice after treatment with CNC018pCH + Doxy were protected against rechallenge with CT26 cells.

Given these promising results, we next examined CNC018pCH in a murine breast cancer (4T1) model (Fig. 6G). Addition of HysA increased tumor penetration by both CNC018pCH and CNC018pHysA compared with CNC018pClyA and CNC018pEmpty (Fig. 6H). In line with our findings for CT26 tumors, CNC018pCH prolonged the survival of murine breast cancer (4T1)-bearing mice, increasing the median survival time from 31 days in the PBS group to 38 days (a 23% increase) in the CNC018pCH treatment group (Fig. S10A–E); however, the therapeutic effect in the 4T1 model was less pronounced than that in CT26, likely due to the limited efficacy of ClyA in 4T1 cells, which may have resulted in weaker tumor cell killing effects and weaker immune activation. Despite this, CNC018pCH treatment did not cause significant changes in body weight or overall appearance (Fig. S10F).

Since 4T1 tumors metastasize spontaneously to the lungs in vivo [32], we explored the potential of CNC018pCH to prevent metastasis. In lung metastatic mice, both CNC018pCH and CNC018pHysA effectively suppressed metastasis to a greater extent than CNC018pEmpty (Fig. 6I and J). These findings are consistent with our in vitro results, highlighting the ability of CNC018pCH to inhibit lung metastasis in the 4T1 model.

### Characterization of immune responses induced by CNC018pCH treatment

**Fig. 7:**
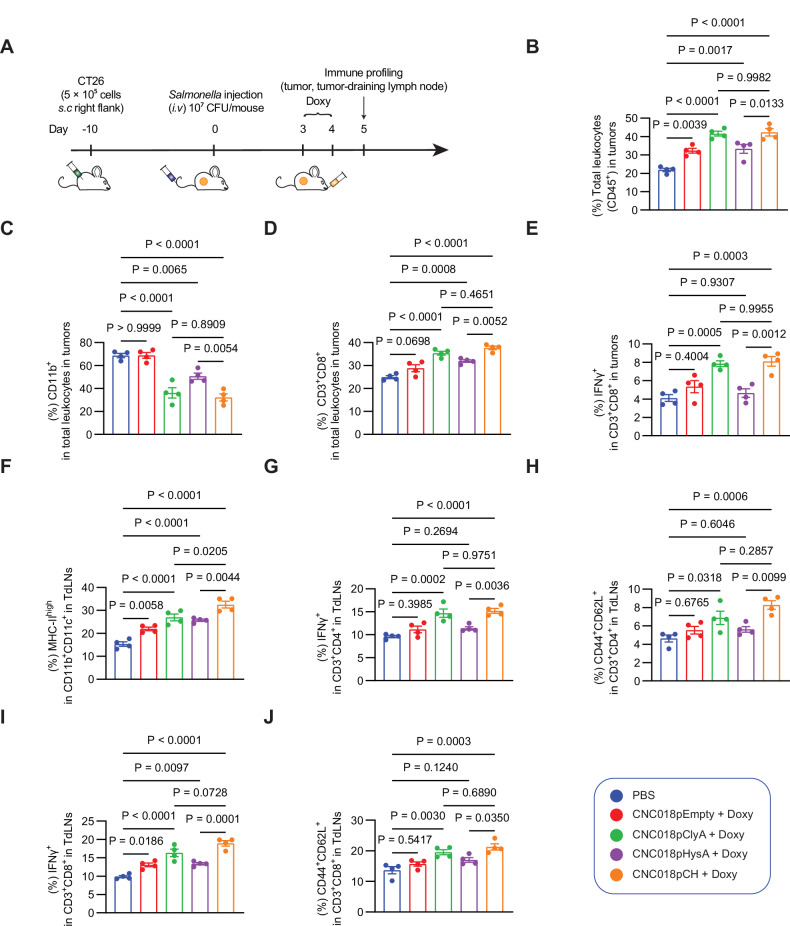
Immune profiling of tumors and TdLNs after treatment with engineered CNC018pCH. **A** Experimental scheme. CT26 cells (5 × 10⁵) were injected subcutaneously into the right flank of mice (n = 4 per group). Once the tumor size reached 120–150 mm³, bacteria (1 × 10⁷ CFU) were injected intravenously, followed by oral administration of 17 mg/kg Doxy every day starting from Day 3 post-infection. Tumors and TdLNs were collected on Day 5 for immune profiling. Frequencies of immune cell populations in tumors: **B** total immune cells (CD45⁺), **C** tumor-infiltrating myeloid cells (CD45⁺ CD11b⁺), **D** total CD8⁺ T cells (CD3⁺ CD8⁺), and **E** activated CD8⁺ T cells (CD3⁺ CD8⁺ IFNγ⁺). Frequencies of immune cell populations in TdLNs: **F** activated dendritic cells (CD11b⁺ CD11c⁺ MHC-II^high^), **G** activated CD4⁺ T cells (CD3⁺ CD4⁺ IFNγ⁺), **H** central memory CD4⁺ T cells (CD3⁺ CD4⁺ CD44⁺ CD62L⁺), **I** activated CD8⁺ T cells (CD3⁺ CD8⁺ IFNγ⁺), and **J** central memory CD8⁺ T cells (CD3⁺ CD8⁺ CD44⁺ CD62L⁺). Please also see flow cytometry gating strategies in Fig. S11. Data are shown as mean ± s.e.m. Statistical significance was determined by one-way ANOVA followed by Tukey’s multiple comparisons test.

To evaluate the immune responses elicited by CNC018pCH, CT26 tumors and tumor-draining lymph nodes (TdLNs) were collected from treated mice and analyzed by flow cytometry (Fig. 7A and Fig. S11). Flow cytometric analysis of tumor samples revealed a marked increase in overall immune cell infiltration (Fig. 7B). Importantly, a shift in the tumor immune landscape was observed, with a decrease in myeloid cells (CD45⁺ CD11b⁺) and a concomitant increase in activated CD8⁺ T cells (Fig. 7C–E), particularly pronounced in the CNC018pCH-treated group.

Consistent with these intratumoral changes, engineered CNC018pCH also promoted robust dendritic cell (DC) activation in TdLNs (Fig. 7F). Notably, the combination of ClyA and HysA synergistically optimized DC activation, which was associated with increased activation of CD4⁺ and CD8⁺ T cells, as well as the generation of central memory subsets in the lymph nodes (Fig. 7G–J).

Together, these results indicate that CNC018pCH effectively remodels the tumor immune microenvironment, shifting it toward a more immunostimulatory state.

## Discussion

In this study, we used attenuated *S. typhimurium* CNC018, engineered (CNC018pCH) to secrete ClyA and HysA under Doxy control, to target and treat various cancer models such as breast and colon cancer. Our in vitro and in vivo experiments showed that CNC018pCH effectively degraded the TME and inhibited metastasis. Furthermore, induction of PANoptosis by the engineered bacteria in a cholesterol-dependent manner triggered inflammatory cell death and suppressed tumor growth, which eventually activated antitumor immunity and established long-term antitumor memory.

Consistent with previous reports, our results support the idea that the ability of HysA to degrade the TME facilitates the spread of *S. typhimurium* within the tumor, leading to improved therapeutic outcomes [20, 21]. Additionally, HysA reduced phosphorylation of metastasis-associated kinases such as PYK2, p70S6K, and ERK1 [33–35], as well as RSKs, although the precise contribution of RSKs to metastatic processes remains incompletely understood [36]. Because HA is a major component of the pericellular glycocalyx of cancer cells, its degradation by HysA likely disrupts HA–CD44 signaling, a key pathway that promotes circulating tumor cell (CTC) survival, immune evasion, and endothelial adhesion during metastatic dissemination [37, 38]. By removing this HA-rich barrier, HysA impairs CTC extravasation and colonization, supporting its anti-metastatic role.

HysA has been reported to reduce tumor interstitial pressure and enhance penetration of chemotherapeutic agents [20, 39], highlighting its potential as a facilitator for more effective drug delivery. Nonetheless, clinical trials using human HysA showed limited therapeutic benefit [40, 41], possibly due to three factors: (1) intravenous HysA may be neutralized by blood components [42]; (2) insufficient tumor-specific localization, especially in HA-rich aggressive tumors [43]; and (3) a fundamental difference between human and bacterial HysA—human HysA produces HA oligomers that can paradoxically promote tumor growth [18], whereas bacterial HysA generates monomers with antitumor effects [44]. These findings indicate bacterial HysA should be a superior candidate for cancer therapy. In this study, *Salmonella*-mediated local secretion of bacterial HysA overcame the limitations observed in clinical trials.

Our data also shed light on the PANoptosis mechanism induced by ClyA, addressing the previously inconsistent findings regarding the role of ClyA in cell death. The effectiveness of ClyA depended on cholesterol levels in cancer cells, which may limit its activity against some cancer types. Increasing expression of cholesterol by cancer cells via pretreatment with MβCD cholesterol improved the efficacy of the construct; however, further optimization of MβCD cholesterol dosing, as well as the administration route, is required.

Although ClyA possesses the potent property to induce immunogenic cell death and release tumor-associated and tumor-specific antigens, resulting in significant tumor suppression [12], ClyA has an important disadvantage: it is not a selective killer. Cholesterol, the key factor for ClyA activity, is overexpressed in some cancers but is also present at significant levels in many normal cell types, which could lead to on-target toxicity if ClyA is administered systemically [13, 45]. Therefore, ClyA is not suitable for standalone clinical application and is better suited for tumor-targeted delivery modalities. For example, in this study, engineered *Salmonella* secreted ClyA locally into tumors to kill cancer cells. With the development of tumor-targeting strategies, ClyA can be considered a potentially safe and effective therapeutic agent.

Importantly, CNC018pCH was able to overcome the immune-privileged status of tumors [46]. Local secretion of HysA and ClyA disrupted both physical and immunological tumor barriers, induced immunogenic cell death, and enhanced T-cell activation and maturation in tumor-draining lymph nodes. This dual mechanism—reducing tumor complexity while stimulating systemic immunity—resulted in robust therapeutic efficacy against both murine and human cancer cell lines. Although *Salmonella* CNC018 retains major lipopolysaccharide (LPS) components, loss of ppGpp signaling broadly down-regulates virulence- and proinflammatory gene expression and alters O-antigen biosynthesis, consistent with an attenuated endotoxin phenotype and reduced systemic inflammatory responses [47–49]. Together, these data confirm CNC018pCH’s high tumor specificity and strong safety profile, supporting its potential for clinical translation.

In summary, we demonstrated that engineered *S. typhimurium* CNC018pCH, capable of secreting ClyA and HysA under the control of Doxy, effectively suppresses metastasis and inhibits tumor growth in multiple cancer models. Furthermore, tumor-free mice treated with CNC018pCH developed memory responses against tumor rechallenge, attributable to the combined activities of ClyA and HysA. These findings provide valuable insights into the mechanisms underlying bacterial cancer therapy and highlight the potential of engineered *S. typhimurium* as a promising therapeutic strategy. However, the study still has some unresolved aspects. The underlying mechanism by which ClyA induces cancer cell death, possibly through ion imbalance following pore formation [13], remains unclear. Similarly, the relationship between CD44-HA signaling and RSK signaling, as observed with HysA, has not been fully elucidated and requires further investigation. In future studies, we aim to delineate the molecular events underlying ClyA-induced PANoptosis, particularly the contribution of pore formation and ion imbalance, by detecting GSDMD N-terminal fragments and visualizing pyroptosis through microscopy. We will also investigate the role of HysA in modulating HA–CD44–RSK signaling and evaluate the therapeutic efficacy of CNC018pCH in orthotopic and metastatic models of breast, colon, and pancreatic cancers to comprehensively assess its translational potential.

## Materials and methods

### Cell lines

Mouse tumor cell lines 4T1 (breast cancer), KPC (pancreatic cancer), CT26 (colon carcinoma) and B16F10 (melanoma), and human tumor cell lines MDA-MB-231 (breast cancer), MCF7 (breast cancer), ASPC-1 (pancreatic cancer), Capan-2 (pancreatic cancer), HCT116 (colon cancer), and HT29 (colon cancer), were obtained from the American Type Culture Collection (ATCC; USA). The authentication was not performed, but mycoplasma contamination was routinely tested. The cells were cultured at 37 °C/5% CO_2_ in Dulbecco’s modified Eagle’s medium (DMEM; Welgene, Korea, LM-001-05) or Roswell Park Memorial Institute 1640 medium (RPMI-1640; Welgene, Korea, LM011-01) supplemented with 10% fetal bovine serum (FBS; Welgene, Korea, S101-07) and 1% penicillin/streptomycin (Welgene, Korea, LS203-01). The cells were detached using 0.05% Trypsin/EDTA (Welgene, Korea, LS-015-01).

### Bacterial strains and transformation

*E. coli* DH5α (Real-biotech, Korea, RH619) was used for cloning and amplification of plasmids. The attenuated *S. typhimurium* strain, CNC018, which lacks two genes responsible for ppGpp synthesis (*relA* and *spoT*) and both Salmonella pathogenicity islands 1 and 2 (*SPI-1* and *SPI-2*) [7], was used for the study. Plasmids were transformed into CNC018 using an electroporator (Bio-Rad, USA) at 1.8 kV, as described previously [23].

### Construction of plasmids

The Doxy-inducible plasmid pJH18, containing the *Ptet* dual promoter (*PtetA/PtetR*), has been described previously [23]. The *clyA* gene fragment, originating from *S. typhi* (GenBank: AL513382), with a Myc-tag (EQKLISEEDL) at the 3′-end, was amplified using the clyA forward (5′-ACTAGTATGACCGGAATATTTGCAGAACAAAC) and clyA-Myc reverse (5′-GAGCTC*TTACAGATCCTCTTCTGAGATGAGTTTTTGTTC*GACGTCAGGAACCTCGAAAAGC) primers, with pJH18-CR [23] as the template. The underlined and italicized sequences indicate the restriction sites (SpeI and SacI) and the Myc-tag, respectively. After digestion with SpeI and SacI, the fragment with the Myc-tag was subcloned into pJH18 downstream of *PtetA*, and the resulting plasmid was named pClyA.

To obtain the *hysA* gene fragment carrying a Flag tag (DYKDDDDK) at the 3′-end, polymerase chain reaction (PCR) was performed using the hysA forward (5′-GGTACCATGGCCACATATAGAATGAAGAAATGG) and hysA-Flag reverse (5′-GTCGAC*TTACTTGTCATCGTCATCCTTGTAATC*TTTAGTTAATTCAAAGTGTACGCCGG) primers, with genomic DNA from *Staphylococcus aureus* ATCC 29213 (BioProject: PRJNA292059) as the template. The underlined and italicized sequences indicate the restriction sites (KpnI and SalI) and the Flag tag, respectively. The fragment was digested with KpnI and SalI restriction enzymes and subcloned downstream of *PtetR* in pJH18 using T4 ligase (Thermo Scientific, USA, EL0011). The resulting plasmid was named pHysA. The Myc-tagged *clyA* gene fragment was digested with SpeI and SacI from pClyA, subcloned into pHysA, and named pCH.

All plasmids were transformed into *E. coli* DH5α, selected on LB agar plates containing 100 μg/mL ampicillin, and sequenced (Macrogen, Korea). CNC018 transformed with the pClyA, pHysA, and pCH plasmids were named CNC018pClyA, CNC018pHysA, and CNC018pCH, respectively. CNC018 carrying the pJH18 plasmid was named CNC018pEmpty.

The *rluc8* gene (encoding Renilla luciferase variant 8) was isolated from pBAD-Rluc8 [50]. PCR was performed using appropriate primers, and both the PCR product and the pJH18 plasmid were digested with the relevant restriction enzymes. The purified products were then ligated using T4 ligase. Subsequently, the ligated plasmid was transformed into DH5α, and colonies showing ampicillin resistance were selected and sequenced to confirm the correct DNA sequence. The plasmid and its transformant were named pRluc8 and CNC018pRluc8, respectively.

### Animal models

Female BALB/c mice, aged 6 weeks, were obtained from Orient BIO (Charles River Laboratories, Korea) and housed under specific pathogen-free conditions throughout the study. All animal experiments were conducted in accordance with the Chonnam National University Animal Research Committee protocol (NIH publication 85–23, revised 1985) to ensure ethical standards. Each mouse was inoculated subcutaneously with 5 × 10⁵ CT26 or 4T1 cells. When the tumors reached approximately 100–120 mm³, mice were injected intravenously with bacterial strains at a dose of 1 × 10⁷ CFU/100 μL. Doxy (17 mg/kg) was administered orally every day, starting from Day 3 post-infection. Tumor size was measured under anesthesia using 2% isoflurane. Tumor volume (mm³) was calculated using the formula: (L × W × H) / 2, where L is the length, W is the width, and H is the height of the tumor (in millimeters). Mice with tumors ≥1500 mm³ were euthanized.

### Western blot analysis to detect payload proteins

After overnight culture, transformed *S. typhimurium* was diluted 100-fold with fresh LB medium containing ampicillin, and further cultured (with agitation) to an optical density at 600 nm (OD_600_) of 0.5–0.7 at 37 °C. Doxy (0–500 ng/mL) was then added at the indicated concentrations, and the culture was continued. After 5 h, the OD_600_ value of each bacterial sample was measured and adjusted to 1 OD_600_ with fresh medium. The samples were centrifuged at 4000 revolutions per minute (rpm) for 5 min to obtain pellets and culture supernatants. The supernatants were filtered through a 0.2-µm membrane to remove any remaining bacteria. Next, 80 µL of bacterial pellet or supernatant was mixed with 20 µL of 5× protein loading buffer (Elpis Biotech, Korea, EBA-1052), boiled for 10 min, and loaded onto a sodium dodecyl sulfate-polyacrylamide gel electrophoresis (SDS-PAGE) gel. Following electrophoresis, proteins were transferred onto a nitrocellulose membrane (GenDEPOT, USA, LC7034-300), which was blocked with 5% skim milk in Tris-buffered saline with 0.1% Tween 20 (TBS-T; Bylabs, Korea, T0161CD) at room temperature for 1 h and then incubated with either anti-Flag (Abcam, UK, ab125243) or anti-Myc (Abcam, UK, ab9106) antibodies (1:1000 dilution) for 2 h. After washing with TBS-T, the membranes were incubated with horseradish peroxidase-conjugated secondary antibodies (Invitrogen, USA, 31460). Finally, the membranes were soaked in Immobilon Western HRP substrate solution (Merck Millipore, Germany, WBKLS0500) and visualized using the ChemiDoc XRS+ machine (Abcam, UK).

To assess whether gene expression affected bacterial integrity, CNC018pCH was treated with 300 ng/mL Doxy, and supernatants were analyzed by western blotting with an anti-DnaK antibody (1:1000 dilution, Enzo Life Sciences, USA, ADI-SPA-880-D).

To estimate the expression of payload proteins in bacteria localized within tumors, 1 × 10^7^ colony-forming units (CFU) of bacteria were injected intravenously into CT26 tumor-bearing mice. Various doses of Doxy were administered orally on Day 3 post-bacterial injection. Tumor tissues were harvested on Day 4 and weighed. These tissues were homogenized in lysis buffer (Pro-prep, intRON, Korea, 17081) and separated (40 µg) by SDS-PAGE. Payload protein levels were analyzed by western blotting with anti-Myc and anti-Flag antibodies.

### Measurement of HysA activity

Bacterial enzyme activity was assessed on HA agar plates as described previously [51]. To prepare these plates, 2 g of agarose (GenDEPOT, USA, A0224-050) was added to a broth containing 120 mL of water and 6 g of tryptic soy broth (Sigma-Aldrich, USA, 22092). After autoclaving and cooling, 40 mL of 1 mg/mL HA (Sigma-Aldrich, USA, 53747), 40 mL of 5% (w/v) bovine serum albumin (GenDEPOT, USA, A0100-010), and 200 µL of 100 mg/mL ampicillin (Duchefa Biochemie, Netherlands, A0104.0010) were added to the agar broth, which was then poured into 10-cm Petri dishes and left overnight at room temperature. Subsequently, 300 ng/mL Doxy was either added or omitted. Bacteria were streaked onto HA agar plates with or without Doxy and incubated at 37 °C. On the following day, 5 mL of acetic acid was poured onto the plates. Bacteria with enzyme activity exhibited transparent zones around colonies.

HysA activity in bacterial supernatants was measured as previously described [52]. Bacteria were prepared as above and centrifuged at 8000 rpm for 2 min to obtain supernatants. Following passage through a 0.2-µm filter, 1 mL of supernatant was mixed with an equal volume of 0.03% HA solution and incubated at 37 °C for 45 min. The reaction was stopped by adding 2.5 mL of acidic albumin solution (79 mM acetic acid, 24 mM sodium acetate, and 0.1% bovine serum albumin). The mixture was incubated for 10 min at room temperature. Turbidity levels were measured at OD_600_ using a spectrophotometer (Shimadzu UV-1280). Enzyme activity was expressed as turbidity reducing units (TRU), and a standard curve was created using recombinant HysA (Sigma, USA, H1136), with TRUs ranging from 1 to 6. TRU per volume was calculated as follows:

$$\begin{array}{ll}\Delta {\rm{OD}}_{600}\,{\rm{Sample}}={\rm{OD}}_{600}\,{\rm{Test}}\,{\mbox{-}}\,{\rm{OD}}_{600}\,{\rm{Blank}}\\\Delta {\rm{OD}}_{600}\,{\rm{Standard}}={\rm{OD}}_{600}\,{\rm{Standard}}\,{\mbox{-}}\,{\rm{OD}}_{600}\,{\rm{Blank}}\;\\ {\rm{TRU}}\; {\rm{Sample}}=({\rm{TRU}}\; {\rm{from}}\; {\rm{standard}}\; {\rm{curve}}\times {\rm{dilution}}\; {\rm{factor}})/{\rm{sample}}\; {\rm{volume}}\end{array}$$

### Measurement of cholesterol in cancer cells

Cholesterol in cancer cells was measured using a cholesterol measurement kit (Sigma-Aldrich, USA, CS0005). Briefly, 1 × 10⁶ cells were mixed with 200 µL of chloroform:isopropanol:IGEPAL® CA-630 (7:11:0.1) in a microhomogenizer. The samples were then centrifuged at 13,000 × *g* for 10 min to remove insoluble material. The organic phase was transferred to a new tube and air-dried at 50 °C for approximately 30 min to remove chloroform. The dried lipids were reconstituted with 200 µL of Assay Buffer and vortexed until the mixture was homogeneous. The samples were then ready for cholesterol measurement using the kit.

### Cholesterol modification in 4T1 cells

For cholesterol enrichment, cells were pretreated with 10 mM methyl-β-cyclodextrin (MβCD; Sigma-Aldrich, USA, C4555-5G) for 60 min and then exposed to 100 μM cholesterol-MβCD inclusion complexes (chol-MβCD) for 12 h [53].

### Cell death analysis

Cells (3 × 10⁵) were seeded in a 12-well plate. After overnight culture, the cells were treated with bacteria at a multiplicity of infection (MOI) of 1 and 300 ng/mL Doxy, and then cultured for a further 16 h. The level of cell death was measured using an Annexin V-PI kit (BD Bioscience, USA, 556547). Briefly, cells with attached bacteria were collected, washed with phosphate-buffered saline (PBS), and stained with Annexin V and propidium iodide (PI) for 15 min. The cells were then analyzed using the BD FACSCanto II machine, and data were processed with FlowJo 9.0 software.

To examine the cell death pathways, cells treated with bacteria were stained for cleaved caspase-3/7, caspase-1, or PI (Immunochemistry, USA, 93 and 97) following the manufacturer’s instructions to detect apoptosis, pyroptosis, and necrosis, respectively. After incubation at 37 °C for 60 min, the cells were washed with the washing buffer (from the kits) and analyzed using the BD FACSCanto II machine. The data were then processed using FlowJo 9.0 software. Autophagy in cancer cells was assessed by western blot using an anti-LC3B antibody (Novus Biologicals, UK, NB600-1384).

### ATP, HMGB1, and LDH assays

The cells (3 × 10⁵) were seeded in a 12-well plate. After overnight culture, the cells were treated with bacteria (1 MOI) and 300 ng/mL Doxy, and cultured for a further 16 h. The cell culture medium was then collected, filtered, and analyzed using an ATP determination kit (ThermoFisher, USA, A22066), western blotting with an HMGB1 antibody (Abcam, UK, ab18256), and in a cytotoxicity assay (Promega, USA, G1780) to estimate the levels of adenosine triphosphate (ATP), high-mobility group box 1 (HMGB1), and lactate dehydrogenase (LDH) release from treated cells.

### Purification of HysA

Bacterial pellets were prepared as described for Western blot analysis. After washing with PBS, the pellet was resuspended (1 OD_600_/mL PBS), protease inhibitor cocktail (GenDEPOT, USA, P3100-010) was added to a final concentration of 1×, and the sample was lysed by sonication. The lysate was then centrifuged at 12,000 rpm for 10 min, and the supernatant was passed through a 0.2-µm filter. HysA was purified from the filtered supernatant using Pierce™ Anti-DYKDDDDK Affinity Resin (Thermo Scientific, USA, A36801).

### HA immunocytochemistry and immunofluorescence microscopy of tumor cells

HA immunocytochemistry was performed as previously described [21]. Briefly, cells (2 × 10⁴) were seeded in an 8-well Nunc® Lab-Tek® II Chamber Slide™ system (Sigma-Aldrich, USA, C7057). When the cells reached 30–40% confluency, the medium was replaced with 200 µL of DMEM supplemented with B-27 (Gibco, USA, 17504044). After 24 h of culture at 37 °C, the slides with cells were fixed in 4% paraformaldehyde (Biosesang, Korea, P2031) and 0.3% Triton X-100 (Sigma-Aldrich, USA, T8787). For HA staining, slides were treated with 0.6% hydrogen peroxide in methanol for 30 min to block endogenous peroxidase activity, and then stained with a biotinylated HA-binding peptide (bHABP; Sigma-Aldrich, USA, H9910) at a final concentration of 5 μg/mL for 2 h at 37 °C. After washing with TBS-T, the slides were stained for 45 min with avidin-labeled horseradish peroxidase (VECTASTAIN® Elite® ABC-Peroxidase Kit; Vector, USA, PK-6200) and visualized using the VECTASTAIN® Elite® ABC-HRP Kit (Vector, USA, PK-6100). Before observation under a light microscope, the slides were counterstained for 2 min with hematoxylin (Ecocell, Korea, HE09-40R).

For tumor staining to assess HA expression and the presence of CNC018, tumors were harvested, fixed for 1 h in 4% paraformaldehyde, and incubated in 30% sucrose overnight at 4 °C before being embedded in Tissue-Tek® O.C.T. Compound (Sakura Finetek, Japan, 25608-930) and stored at −80 °C. The next day, the tissue was sectioned at a depth of 6 μm. The slides were dried, fixed in cold acetone, and incubated for 2 h at 37 °C with bHABP (final concentration, 5 mg/mL) and an anti-*Salmonella* antibody (1:100; Abcam, UK, AB35156). Streptavidin-PE (Vectorlabs, USA, SA-5207-1) and Alexa Fluor™ 488 anti-rabbit antibodies (Invitrogen, USA, A-11006) were used to visualize HA and *S. typhimurium* by fluorescence microscopy (Zeiss Observer II), with DAPI used for nuclear staining. Tiling was performed at 5× magnification.

### Measurement of HA levels

Cells (3 × 10⁵) were seeded in a 12-well plate. After 24 h, the cell culture medium was collected, filtered, and analyzed using the Hyaluronan Quantikine ELISA Kit (R&D Systems, USA, DHYAL0).

### Flow cytometry in tumor cells

Tumor cells (10⁶ cells) were detached using 0.05% trypsin-EDTA (Welgene, Korea, LS-015-01) and stained with anti-vimentin (V9) Alexa Fluor® 647 (Santa Cruz, USA, sc-6260 AF647), or anti-E-cadherin Alexa Fluor® 488 (Cell Signaling, USA, 3199S) antibodies, combined with an anti-CD44 antibody PE (BioLegend, USA, 103024), all diluted at 1:200. The stained cells were washed with FACS buffer (DPBS + 1% FBS) and analyzed using a FACSCanto II flow cytometer (Becton Dickinson, USA) to obtain mean fluorescence intensity (MFI) values.

### Invasion assay

Cells (3 × 10⁴/well) were seeded in 24-well Matrigel invasion chambers (Corning, 354483) and treated for 24 h with purified HysA (100 units/mL). Invaded cells were fixed in 4% paraformaldehyde and 100% methanol (5 min each). The fixed cells were stained with Crystal Violet solution (Sigma-Aldrich, USA, V5265-500ML) and counted under a light microscope.

### Knockout of *HAS2* gene using CRISPR-Cas9

The *HAS2* gene was deleted using the CRISPR-Cas9 KN2.0 non-homology-mediated method obtained from Origene Technologies Inc (USA). Using a simplified protocol, 3 × 10⁵ MDA-MB-231 cells were seeded in 6-well plates and transfected with a commercialized HAS2 guide RNA (Santa Cruz, USA, sc-401032) and a donor cassette (EF1a-GFP-P2A-Puro; Origene, USA, KN501741D) using TurboFectin (Origene, USA, TF81001). The transfected cells were then selected in a medium containing 5 μg/mL puromycin (Sigma-Aldrich, USA, P9620-10ML). Knockout of *HAS2* was confirmed by western blot analysis with an anti-HAS2 antibody (Santa Cruz Biotechnology, USA, sc-514737).

### Optical bioluminescence imaging of Rluc8 expression

Initially, mice were inoculated subcutaneously with 5 × 10⁵ CT26 cells. When the tumor reached 150 mm³, 1 × 10⁷ CFU CNC018pRluc8 were injected. At 3 days post-injection, the mice were orally administered Doxy. Bioluminescence signals were evaluated using an In Vivo Imaging System (IVIS) before and after 12 h of Doxy induction at doses of 1.7 mg/kg or 17 mg/kg. To assess the bioluminescence signal, coelenterazine (Biotum, USA, 10110-1) dissolved in PBS was injected intravenously at a dose of 0.7 mg/kg body weight in a final volume of 200 µL. Imaging signals within the regions of interest were quantified as units of total flux (p/s), as described previously [54].

### Quantification of tumor-localizing bacteria

CT26 tumor cells (5 × 10⁵) were implanted subcutaneously into BALB/c mice. Upon reaching approximately 150 mm³, CNC018pCH bacteria (1 × 10⁷ CFU) were injected intravenously on Day 0. Doxy (0, 1.7, 17, 34, and 85 mg/kg body weight) was administered orally (daily, starting from Day 3). Tumors were excised on Day 4, weighed, and homogenized in 1 mL of PBS. The homogenate was then diluted 10⁴–10⁶-fold with PBS and inoculated onto LB agar plates containing ampicillin. After overnight incubation at 37 °C, bacterial colonies were counted and reported as CFU/g tissue.

### Staining for lung metastasis

Pulmonary metastases were identified using India ink staining [55]. After intra-tracheal injection of 1.5 mL of India ink (15% India ink, 85% water, and three drops of NH₄OH in 100 mL; Becton Dickinson, USA, 261194), the lungs were washed with Feket’s solution (300 mL 70% ethanol, 30 mL 37% formaldehyde, and 5 mL glacial acetic acid) and then placed in fresh Feket’s solution overnight. White tumor nodules in the black-stained lungs were identified and counted.

### Immune profiling analysis

Solid tumors and tumor-draining lymph nodes (TdLNs) were collected from mice on day 5 after bacterial treatment. Samples were digested with collagen type IV (1 mg/mL; Roche, Switzerland, 11088866001) and DNase I (50 μg/mL; Roche, 11284932001) at 37 °C for 30 min, then passed through 100-μm cell strainers (SPL Life Sciences, Korea, 93100) to obtain single-cell suspensions. Next, 2 mL of each sample was mixed with an equal volume of 1× lysis buffer (QIAGEN, Germany, 31014) and incubated for 4 min at 37 °C to lyse red blood cells. The cells were then filtered through 40-μm cell strainers (SPL Life Sciences, Korea, 93040). Fc receptors were blocked with anti-mouse CD16/CD32 antibody (Biolegend, USA, 101320) for 10 min at 4 °C. Without washing, cells were stained for 1 h at 4 °C with fluorochrome-conjugated antibodies (listed in Supplementary Table S1). After washing with FACS buffer, cells were fixed and permeabilized using the BD Cytofix/Cytoperm™ kit (BD Biosciences, USA, 554714), followed by staining with IFNγ antibody. Fluorescence signals were measured by flow cytometry (CytoFLEX LX, Beckman Coulter, USA), and data were analyzed using FlowJo 9.0 software.

### Statistical analysis

No formal sample size calculation was performed; the number of biological replicates for in vitro experiments and the number of animals per group in in vivo experiments were determined based on prior experience and feasibility. No samples or animals were excluded from the analysis. Samples and animals were assigned to groups based on availability, without formal randomization. Investigators were aware of group allocation during both experimentation and outcome assessment (no blinding). Statistical analyses were performed using GraphPad Prism version 9 (GraphPad Software, USA). All quantification data are presented as the mean ± standard error of the mean (s.e.m.). Statistical significance was assessed using Student’s t-test, one-way ANOVA, two-way ANOVA, or log-rank (Mantel–Cox) test as appropriate, as described in the figure legends. Approximate normal distribution of the data was assumed; no formal normality tests were performed. Variance was considered comparable across groups, without formal testing. All tests were two-sided, and P-values < 0.05 were considered statistically significant.

## Supplementary information

Supplemental Information

Supplementary Original Western Blots

## Data Availability

The datasets generated during and/or analyzed during the current study are available from the corresponding author on reasonable request.
