# Supplementary material for: Salmonella typhimurium co-expressing cytolysin A and hyaluronidase suppresses tumor growth and metastasis
Source: Cell Death Discov. 2026 Jan 2;12:75. doi: 10.1038/s41420-025-02897-9 (PMC12859083; doi:10.1038/s41420-025-02897-9)
Supplement: Supplementary file 1 — Supplemental Information [file 41420_2025_2897_MOESM1_ESM.docx]

Supplementary Tables and Supplementary Figures for

***Salmonella typhimurium* co-expressing cytolysin A and hyaluronidase**

**suppresses tumor growth and metastasis**

Khuynh Van Nguyen *et al*.

***Corresponding author:**

Jung-Joon Min ([jjmin@jnu.ac.kr](mailto:jjmin@jnu.ac.kr))

Yeongjin Hong ([yjhong@chonnam.ac.kr](mailto:yjhong@chonnam.ac.kr))

**Supplementary tables**

**Table S1.** Antibodies used for the study

| **Antibody name** | **Company** | **Catalog No.** | **Application** |
| --- | --- | --- | --- |
| Anti-DnaK | Enzo Life Sciences | ADI-SPA-880D | WB |
| Alexa Fluor™ 555 anti-rabbit | Invitrogen | A-31572 | IF |
| Anti-CD44 | Abcam | Ab157107 | WB |
| Anti-CD44 antibody PE | BioLegend | 103024 | Flow |
| Anti-E-cadherin | Cell Signaling | 3195S | WB |
| Anti-E-cadherin Alexa Fluor® 488 | Cell Signaling | 3199S | Flow |
| Anti-Flag tag | Abcam | Ab1162 | WB |
| Anti-HAS2 | Santa Cruz | Sc-514737 | WB |
| Anti-HMGB1 | Abcam | Ab18256 | WB |
| Anti-LC3B | Novus Biologicals | NB600-1384 | WB |
| Anti-Myc tag | Abcam | Ab9132 | WB |
| Anti-RSK1 | Cell Signaling | 9333S | WB |
| Anti-RSK2 | Cell Signaling | 5528S | WB |
| Anti-Salmonella | Abcam | Ab35156 | IF |
| Anti-vimentin (V9) | Santa Cruz | Sc-6260 | WB |
| Anti-vimentin (V9) Alexa Fluor® 647 | Santa Cruz | Sc-6260 AF647 | Flow |
| FAM-FLICA® Caspase-1 (YVAD) Assay Kit | Immunochemistry | 97 | Flow |
| FAM-FLICA® Caspase-3/7 Assay Kit | Immunochemistry | 93 | Flow |
| FITC Annexin V Apoptosis Detection Kit I | BD Biosciences | 556547 | Flow |
| Goat anti-Rabbit IgG (H+L) secondary antibody, HRP | Invitrogen | 31460 | WB |
| Human/Mouse Phospho-RSK1 (S221)/RSK2 (S227) Antibody | R&D Systems | MAB892-SP | WB |
| Hyaluronic Acid Binding Protein−Biotin bovine (bHABP) | Sigma-Aldrich | H9910 | IF, IHC |
| Streptavidin-PE | Vectorlabs | SA-5207-1 | IF |
| LIVE/DEAD™ Fixable Aqua Dead Cell Stain Kit | Invitrogen | L34957 | Flow |
| Pacific Blue CD45 Ab | Biolegend | 103126 | Flow |
| APC/Cy7 anti-mouse CD11b Ab | Biolegend | 101226 | Flow |
| PerCP/Cy5.5 anti-mouse MHC-II Ab | Biolegend | 107626 | Flow |
| PE/Cy7 anti-mouse CD11c Ab | Biolegend | 117318 | Flow |
| FITC anti-CD3 monoclonal Ab | eBioscience™ | 11-0038-42 | Flow |
| PE anti-mouse CD4 Ab | Biolegend | 100511 | Flow |
| BV605 anti-mouse CD8a Ab | Biolegend | 100744 | Flow |
| BV650 anti-mouse CD44 Ab | Biolegend | 103049 | Flow |
| APC/Cy7 anti-mouse IFN-γ Ab | Biolegend | 505850 | Flow |
| Alexa Fluor 700 anti-mouse CD62L Ab | Biolegend | 104426 | Flow |
|  |  |  |  |

**Supplementary figures**


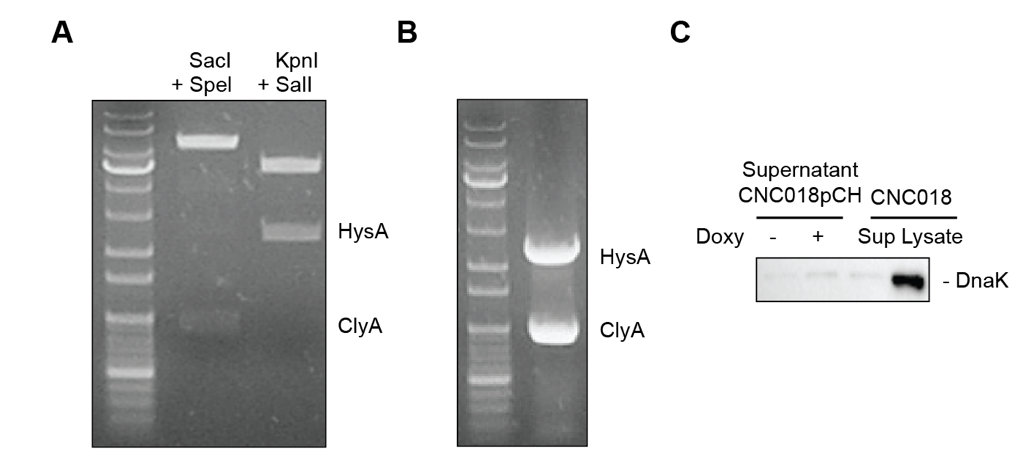


**Figure S1. Functional characterization of CNC018pCH bacteria.**

(**A**) Restriction mapping of pCH. The plasmid was digested with SacI and SpeI to target the *clyA* gene under the *PtetA* promoter (lane 1), or with SalI and KpnI to target the *hysA* gene under the *PtetR* promoter (lane 2). (**B**) PCR analysis of pCH. Gene fragments were amplified using primer mixtures containing clyA forward and clyA-Myc reverse primers specific for *clyA*, and hysA forward and hysA-Flag reverse primers specific for *hysA*, using pCH as the template. (**C**) Western blot analysis of DnaK in CNC018pCH culture supernatant. Bacteria were treated with (+) or without (-) 300 ng/mL Doxy. After 5 h of culture, supernatants were analyzed by western blot with an anti-DnaK antibody. Sup: bacterial supernatant; Lysate: bacterial lysate.


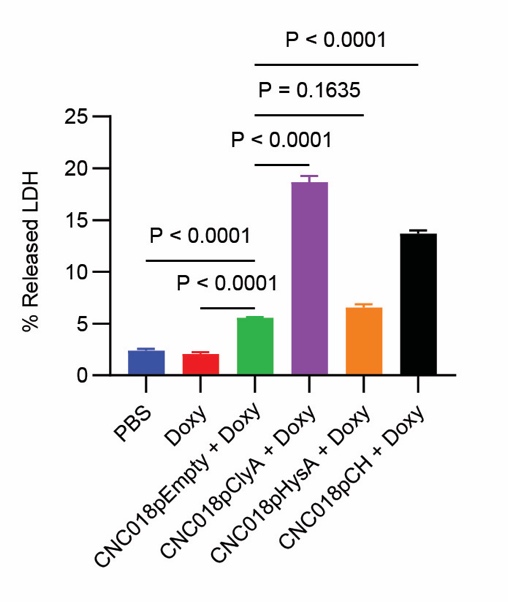


**Figure S2. Release of LDH from tumor cells treated with ClyA-secreting bacteria.**

CT26 tumor cells (3 × 10⁵) were treated with bacteria (1 MOI) plus 300 ng/mL Doxy. After 16 h, LDH levels in the culture supernatant were measured using a cytotoxicity assay kit.

**
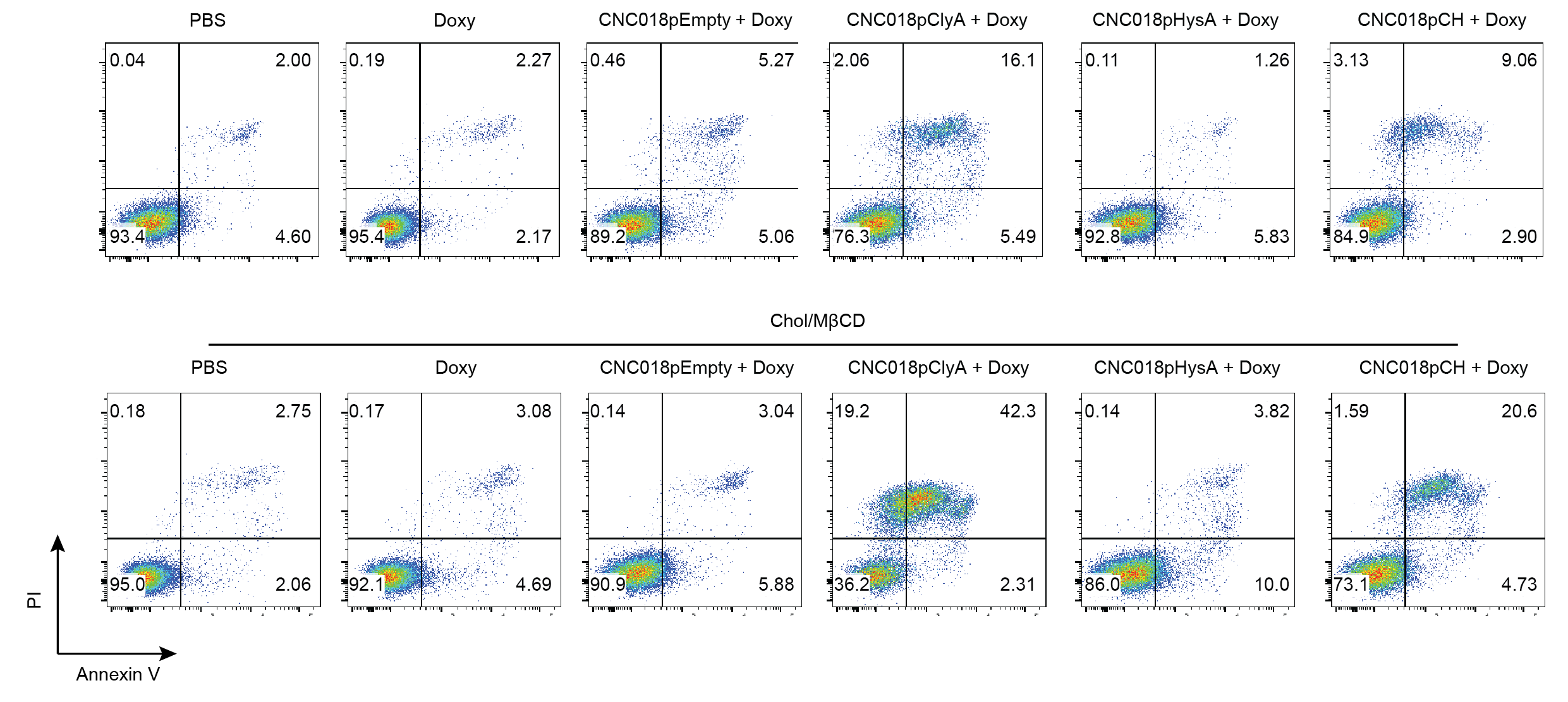
**

**Figure S3. Cytotoxicity of CNC018pCH against 4T1 tumor cells supplemented with cholesterol.**

Cholesterol-supplemented 4T1 tumor cells were incubated with bacteria (MOI 1) plus 300 ng/mL Doxy for 16 h. Cells were then stained with Annexin V-PI and analyzed by flow cytometry.

**
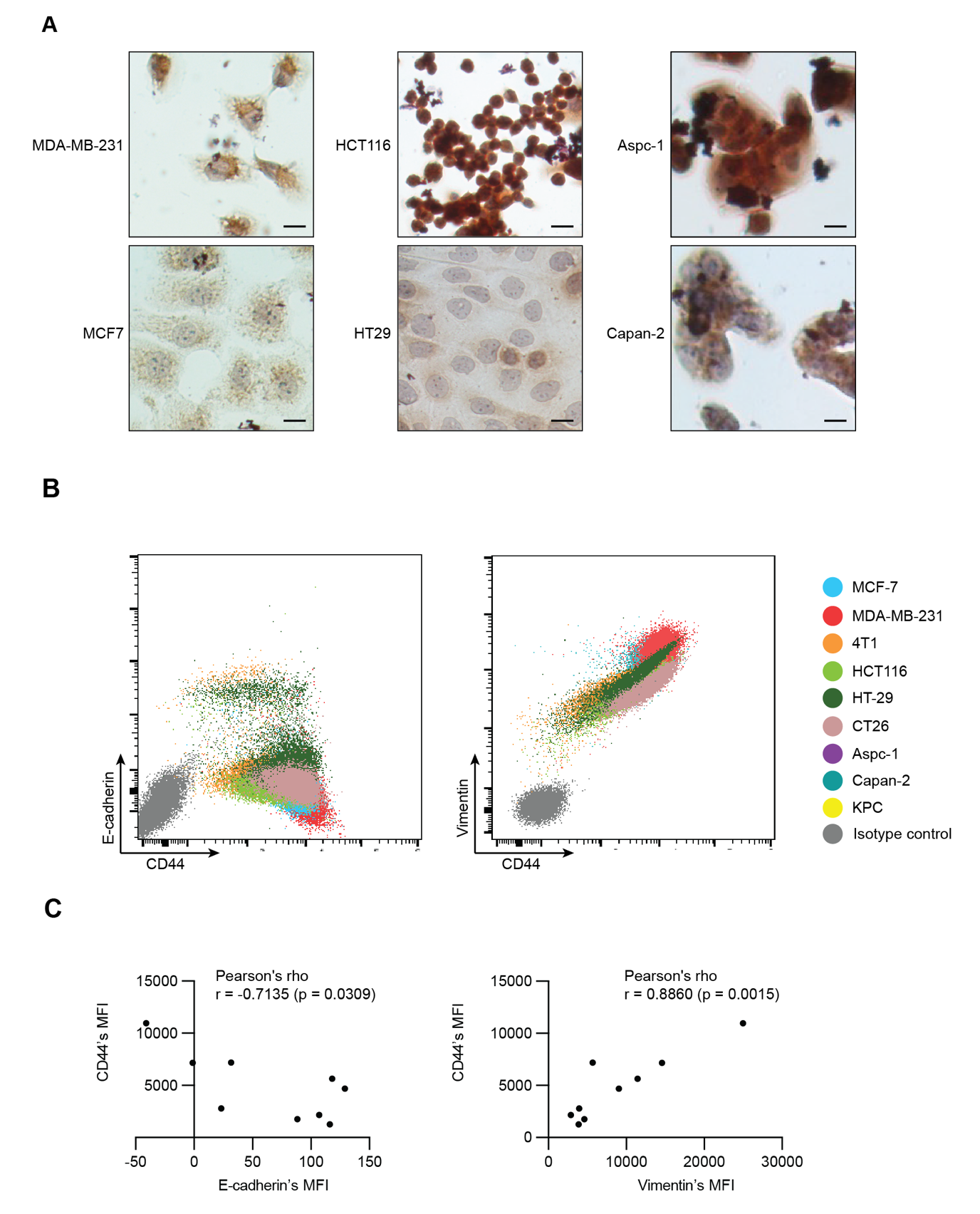
**

**Figure S4. HA levels, and correlation between CD44 and E-Cadherin/vimentin expression, in tumor cells.**

(**A**) Immunocytochemistry was performed using bHABP to detect HA in human tumor cells (brown staining). Images were captured at 400× magnification. Scale bar: 10 µm. (**B**) Correlation between CD44 and vimentin or E-cadherin in tumor cells. The cells were stained with antibodies specific to vimentin or E-cadherin and CD44, and fluorescence levels were measured using flow cytometry. (**C**) Mean fluorescence intensities (MFIs) of CD44 staining in (**B**) were plotted against those of vimentin or E-cadherin staining. Correlation was assessed using Pearson’s rho test.


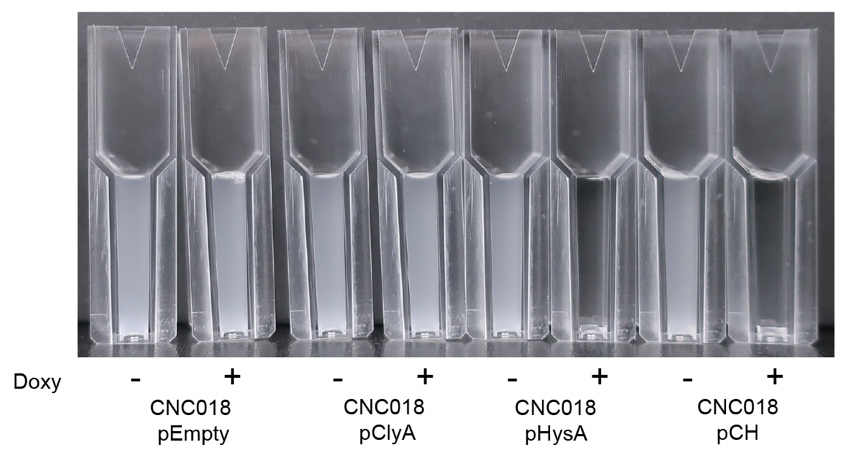


**Figure S5. HysA activity in bacterial supernatant.**Bacteria were treated with (+) or without (-) 300 ng/mL Doxy. After 5 h of culture, the bacterial supernatants were analyzed following the HA degradation reaction.

**
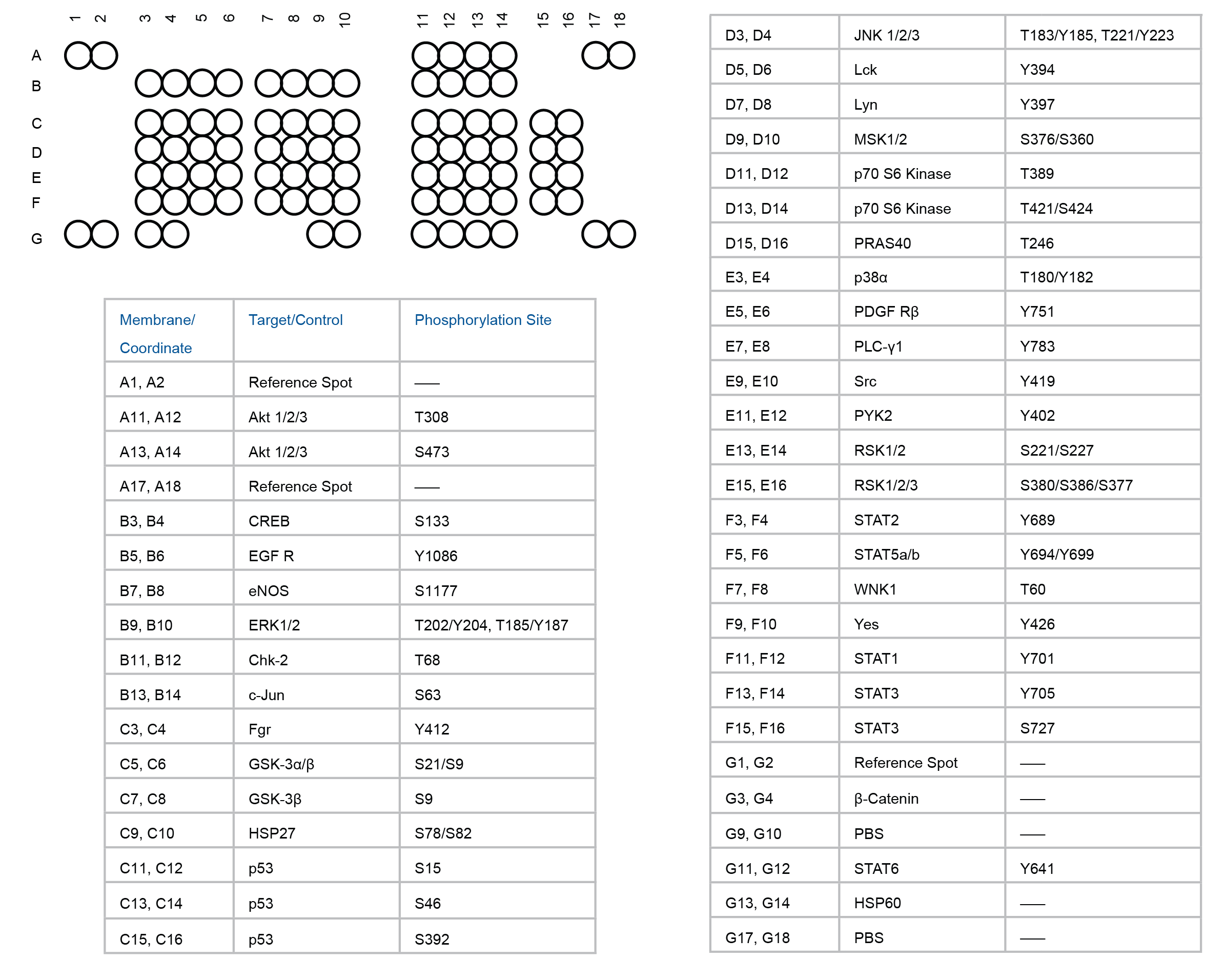
**

**Figure S6. Phospho-kinase array.**Human phospho-kinase array coordinate map.


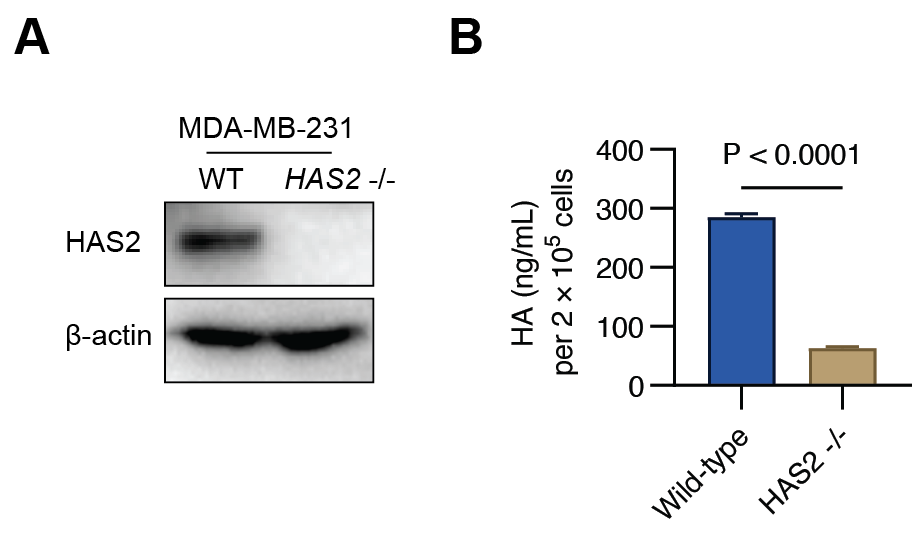


**Figure S7. Knockout of the *HAS2* gene in MDA-MB-231 cells.**The *HAS2* gene in MDA-MB-231 cells was deleted using the CRISPR/Cas9 system. (**A**) Western blot analysis with an anti-HAS2 antibody was performed to confirm deletion of the HAS2 protein. (**B**) HA levels in the cell culture supernatant were measured using an ELISA assay to assess the impact of *HAS2* knockout on HA secretion.


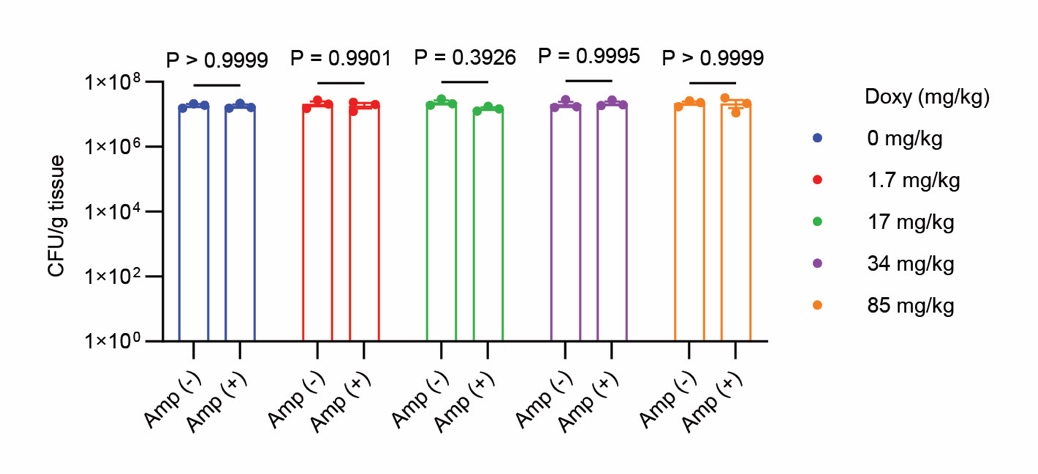


**Figure S8. Assessment of plasmid stability following Doxy induction.**

CT26 cells (5 × 10⁵) were injected subcutaneously into the right flank of mice (three per group). When tumors reached a volume of 100–120 mm³, mice were injected with bacteria (10⁷ CFU) and orally administered the indicated doses of Doxy on Day 3. On day 4, the number of CNC018pCH bacteria within tumors was quantified on LB agar plates with [amp (+)] or without [amp (–)] ampicillin.

**
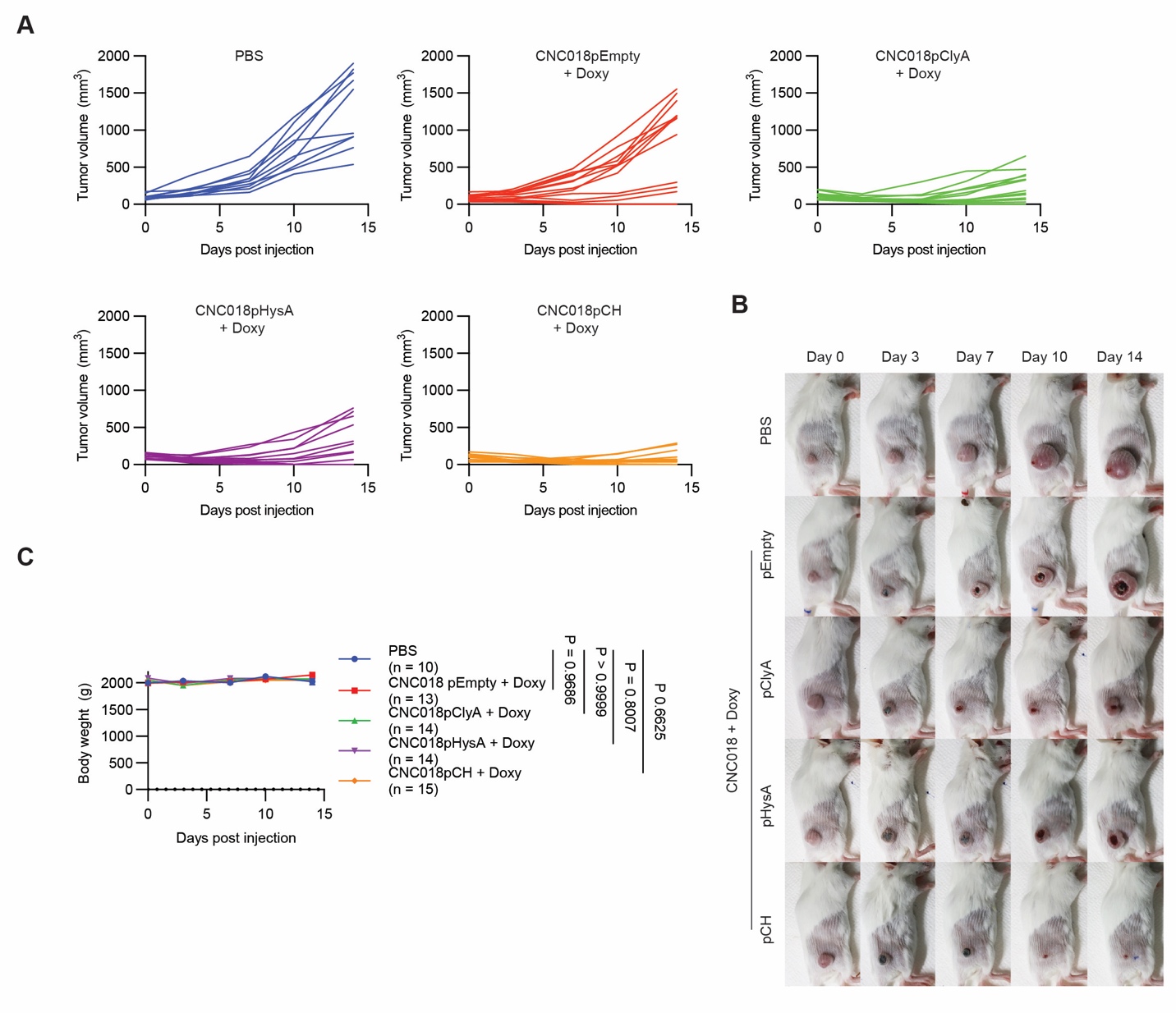
**

**Figure S9. Tumor growth curves for individual mice carrying CT26 tumors.**(**A**) The CT26 model. Data are representative of two independent experimental replicates. (**B**) Representative images of CT26 tumor-bearing mice post-treatment. (**C**) Body weight after treatment.

**
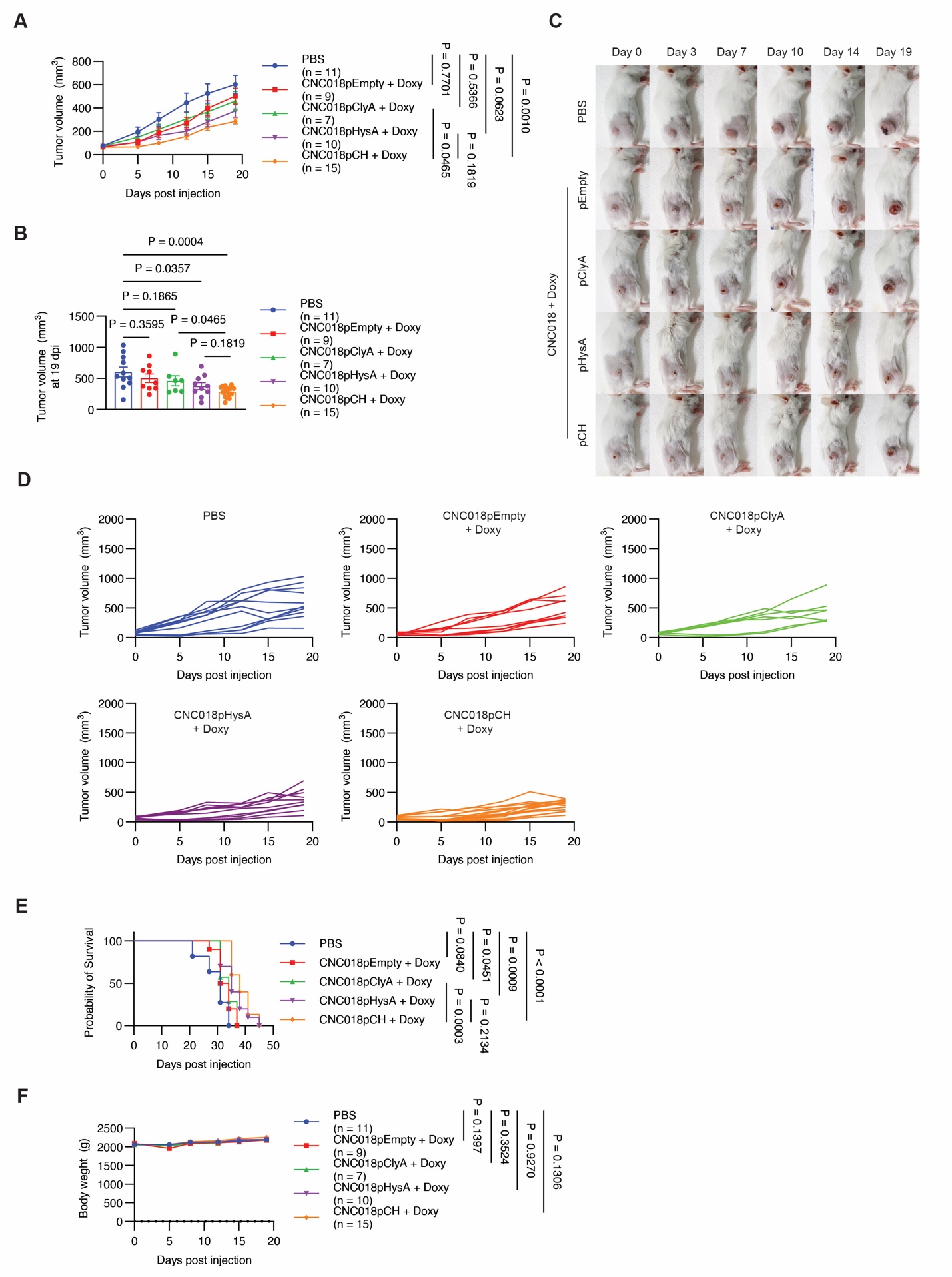
**

**Figure S10. Tumor growth curves for individual mice bearing 4T1 tumors.**

(**A**) Tumor growth curves for 4T1 tumor-bearing mice. Mice bearing subcutaneous 4T1 tumors were treated as described in **Figure 6A**. Data are representative of two independent experimental replicates. (**B**) Tumor volume at Day 19 post-treatment. (**C**) Representative images of 4T1 tumor-bearing mice after treatment. (**D**) Tumor growth curves for individual mice. (**E**) Kaplan–Meier survival curves of 4T1 tumor-bearing mice (log-rank (Mantel–Cox test)). (**F**) Body weight after treatment.

**
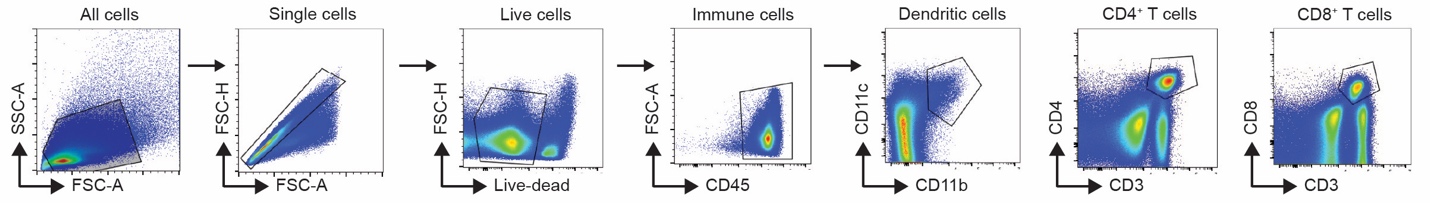
**

**Figure S11. Flow cytometry gating strategies.**

Tumors and TdLNs were collected from CT26 tumor-bearing mice following bacterial injection and Doxy treatment, and immune cells were analyzed by flow cytometry. Gating strategy using the fluorescence minus one (FMO) control to set gates.
