# Supplementary figures and images for: Salmonella typhimurium co-expressing cytolysin A and hyaluronidase suppresses tumor growth and metastasis
Source: Cell Death Discov. 2026 Jan 2;12:75. doi: 10.1038/s41420-025-02897-9 (PMC12859083; doi:10.1038/s41420-025-02897-9)

Figure 2

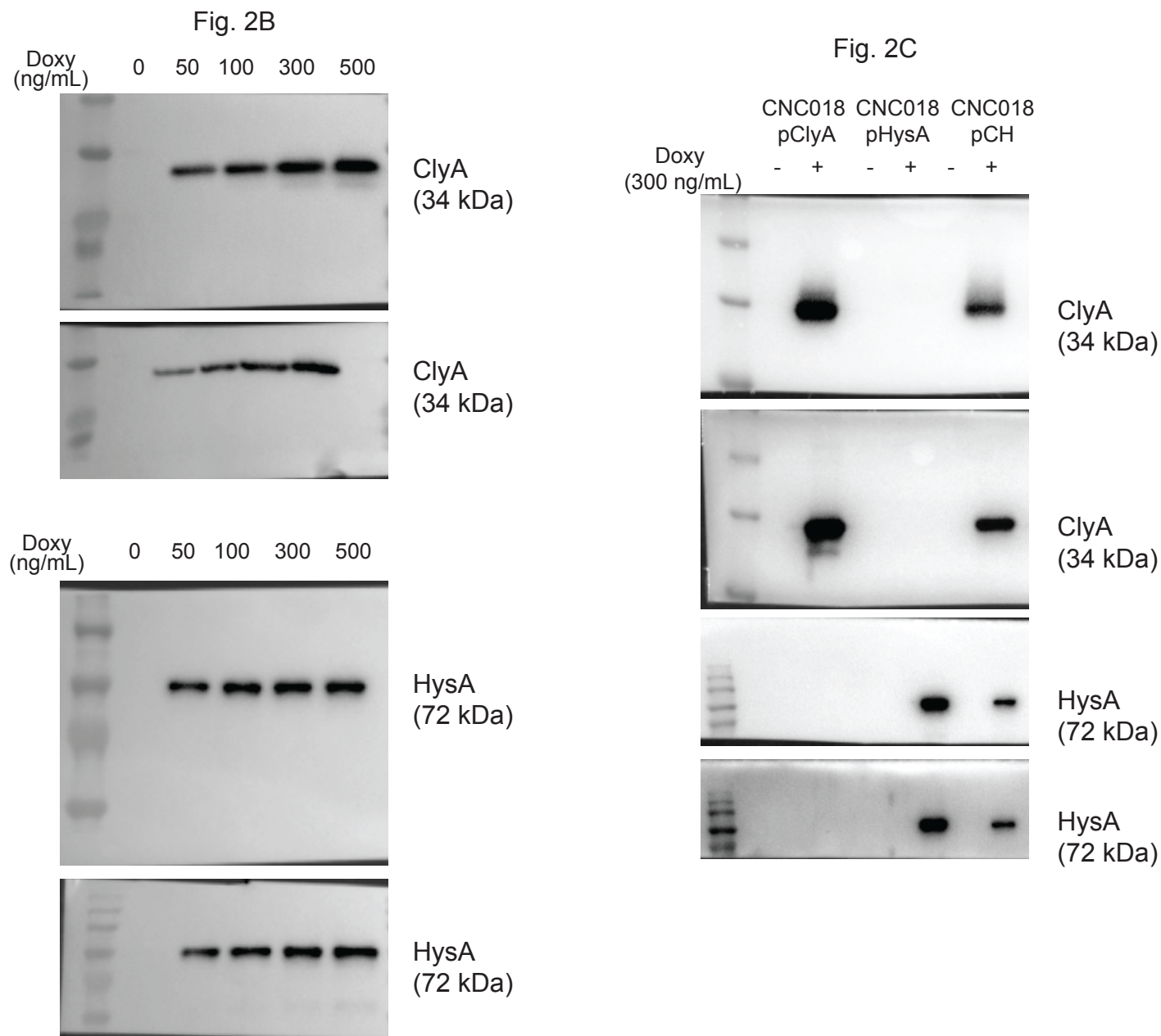

Figure 3

Fig. 3B

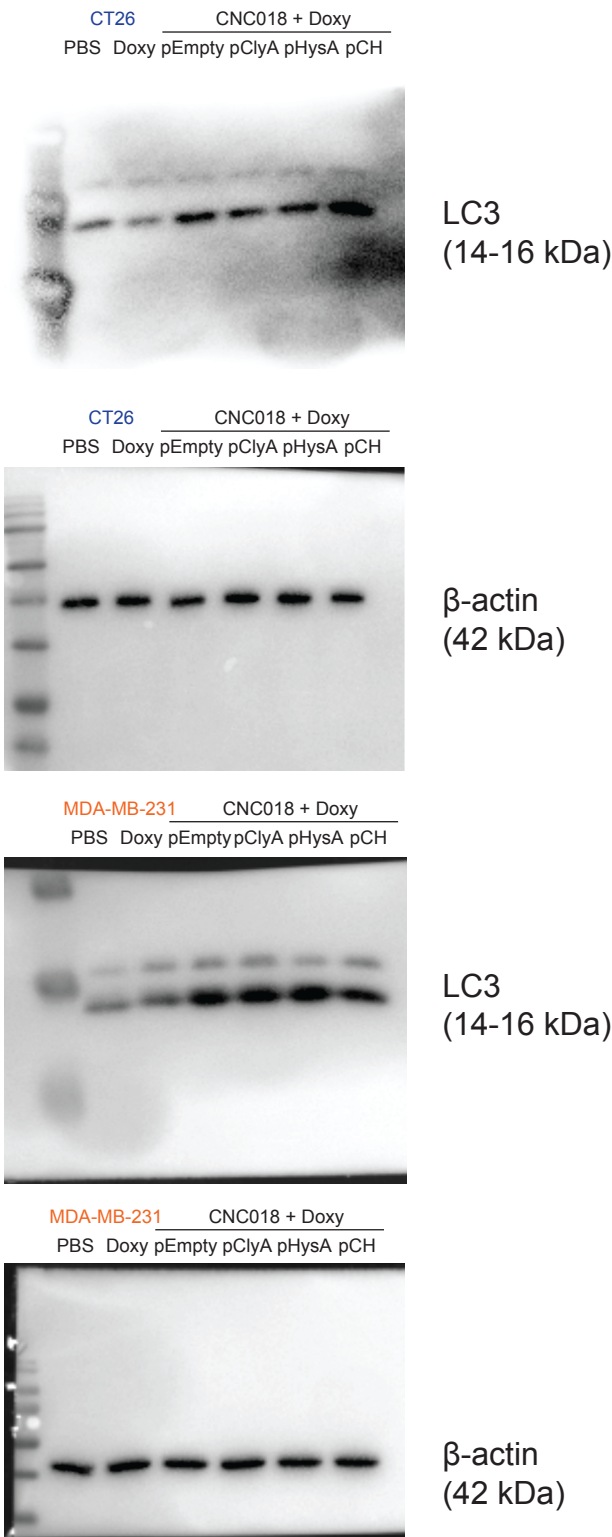

Fig. 3C

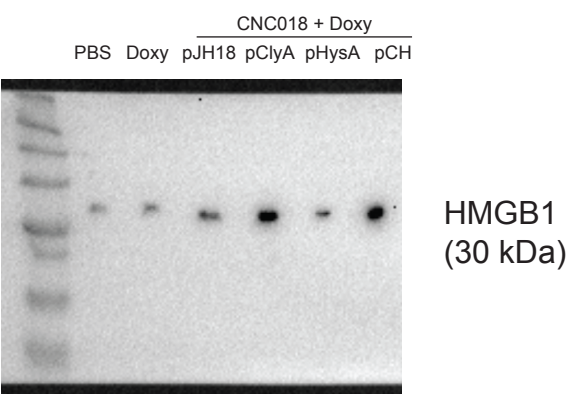

Figure 4

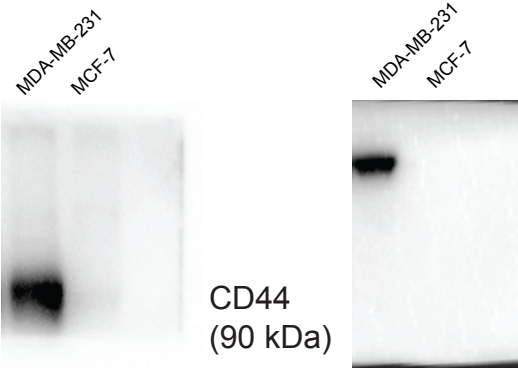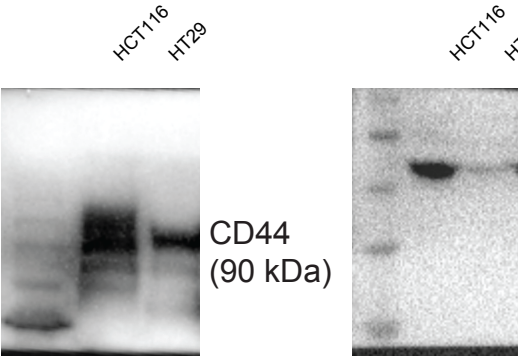

Fig. 4B

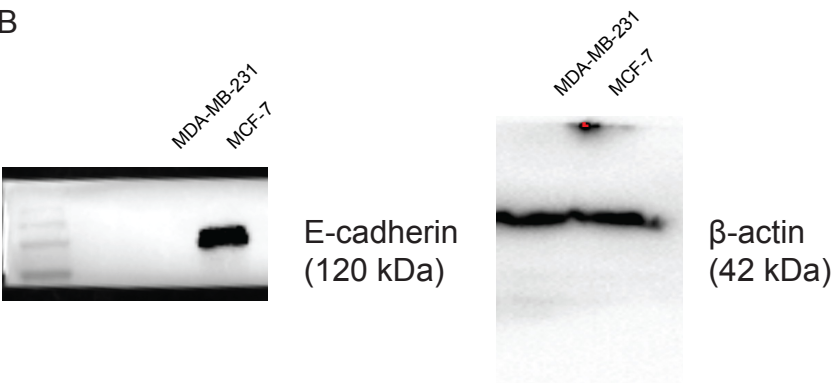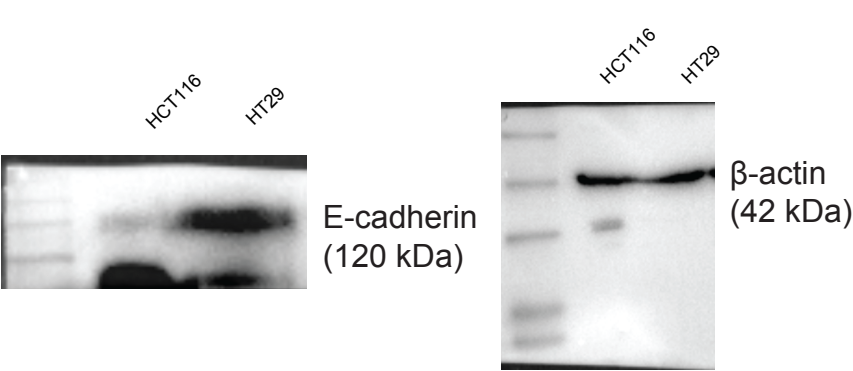

Fig. 4I

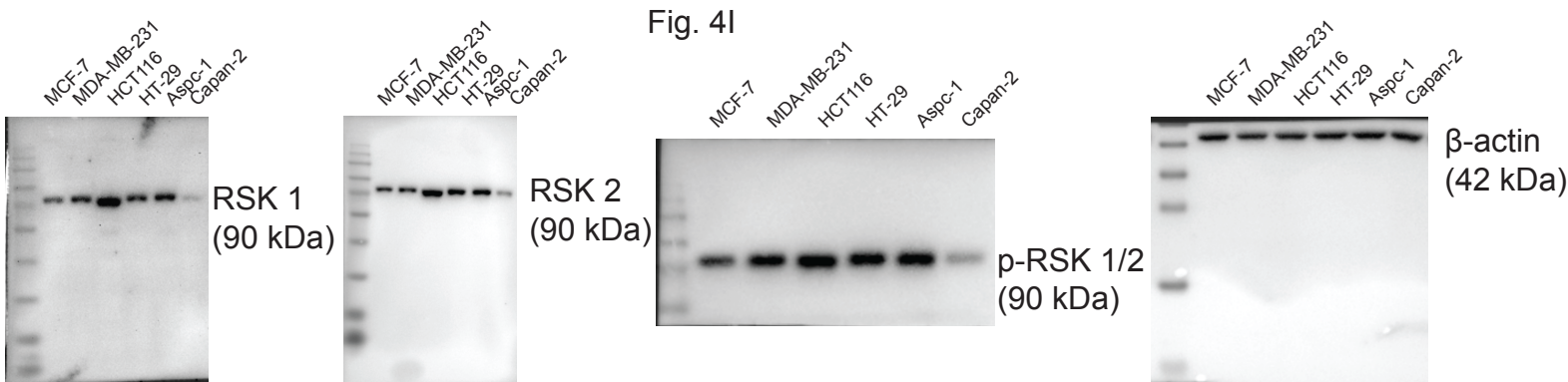

Fig. 4J

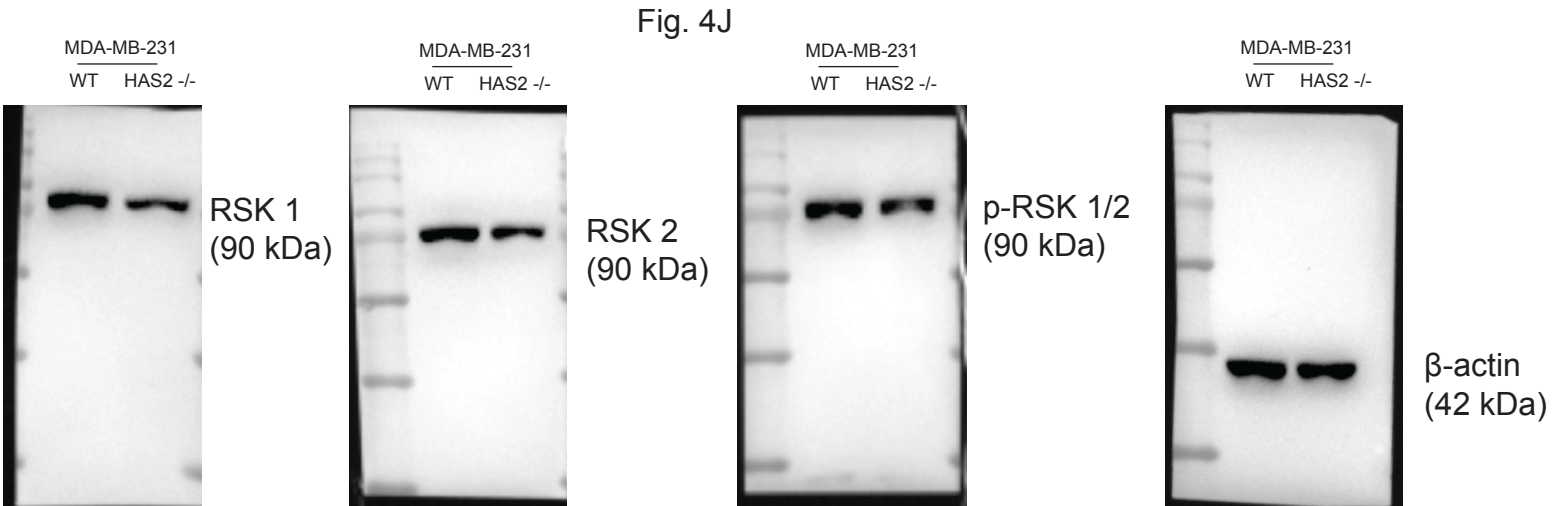

Figure 5

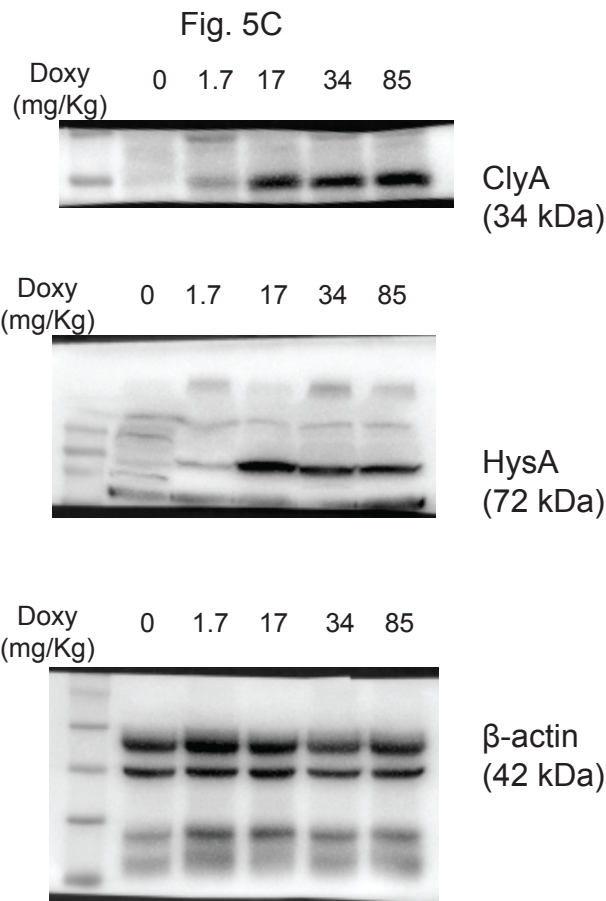

Supplement: Supplementary file 2 — Supplementary Original Western Blots [file 41420_2025_2897_MOESM2_ESM.pdf]
